# Herpes Simplex Virus type 1 infects Langerhans cells and the novel epidermal dendritic cell, Epi-cDC2s, via different entry pathways

**DOI:** 10.1371/journal.ppat.1009536

**Published:** 2021-04-27

**Authors:** Kirstie M. Bertram, Naomi R. Truong, Jacinta B. Smith, Min Kim, Kerrie J. Sandgren, Konrad L. Feng, Jason J. Herbert, Hafsa Rana, Kevin Danastas, Monica Miranda-Saksena, Jake W. Rhodes, Ellis Patrick, Ralph C. Cohen, Jake Lim, Steven L. Merten, Andrew N. Harman, Anthony L. Cunningham

**Affiliations:** 1 Centre for Virus Research, The Westmead Institute for Medical Research, Westmead, Australia; 2 The Westmead Clinical School, Faculty of Medicine and Health, The University of Sydney, Westmead, Australia; 3 The School of Mathematics and Statistics, Faculty of Science, The University of Sydney, Camperdown, Australia; 4 Department of Surgery, University of Sydney and The Children’s Hospital at Westmead, Westmead, Australia; 5 Department of Surgery, Westmead Private Hospital, Westmead, Australia; 6 Department of Surgery, Macquarie University Hospital, Macquarie Park, Australia; 7 The School of Medical Sciences, Faculty of Medicine and Health, University of Sydney, Westmead, Australia; University of Wisconsin-Madison, UNITED STATES

## Abstract

Skin mononuclear phagocytes (MNPs) provide the first interactions of invading viruses with the immune system. In addition to Langerhans cells (LCs), we recently described a second epidermal MNP population, Epi-cDC2s, in human anogenital epidermis that is closely related to dermal conventional dendritic cells type 2 (cDC2) and can be preferentially infected by HIV. Here we show that in epidermal explants topically infected with herpes simplex virus (HSV-1), both LCs and Epi-cDC2s interact with HSV-1 particles and infected keratinocytes. Isolated Epi-cDC2s support higher levels of infection than LCs *in vitro*, inhibited by acyclovir, but both MNP subtypes express similar levels of the HSV entry receptors nectin-1 and HVEM, and show similar levels of initial uptake. Using inhibitors of endosomal acidification, actin and cholesterol, we found that HSV-1 utilises different entry pathways in each cell type. HSV-1 predominantly infects LCs, and monocyte-derived MNPs, via a pH-dependent pathway. In contrast, Epi-cDC2s are mainly infected via a pH-independent pathway which may contribute to the enhanced infection of Epi-cDC2s. Both cells underwent apoptosis suggesting that Epi-cDC2s may follow the same dermal migration and uptake by dermal MNPs that we have previously shown for LCs. Thus, we hypothesize that the uptake of HSV and infection of Epi-cDC2s will stimulate immune responses via a different pathway to LCs, which in future may help guide HSV vaccine development and adjuvant targeting.

## Author summary

Here we describe the first interactions of herpes simplex virus type 1 (HSV-1) with the human immune system as it enters the human genital skin. It was previously thought that this initial interaction of HSV-1 was only with ‘Langerhans cells’ (LCs) but we describe another important interaction with a new immune cell, Epi-cDC2s, which can also be infected by HIV and much more efficiently than LCs. Thus, there are now two of these types of immune cells resident in human epidermis. Therefore the way in which sexually transmitted and skin infecting viruses establish infection needs to be re-examined, including for HSV. We compared how these two cell types interacted with HSV-1 and showed Epi-cDC2s are more susceptible to HSV-1 infection than LCs and take up the virus via a different entry pathway. We speculate these cells may then carry HSV fragments to the dermis and then to lymph nodes to stimulate a protective immune response. These findings suggest similar routes may apply for chickenpox and cowpox viruses which also enter via the skin. Studying this pathway to establishment of natural immunity to HSV, may help guide the development of a successful vaccine.

## Introduction

Herpes Simplex Virus type 1 (HSV-1) causes orofacial and genital herpes as well as encephalitis, neonatal herpes, and keratitis and blindness. HSV-2 also causes genital herpes and predisposes to HIV acquisition. A vaccine is needed for both HSV-1 and -2. Elucidation of the mechanisms of initiation of the immune response after mucosal/skin infection will help determine how to target antigen presenting cells with antigens and adjuvants to stimulate all relevant modes of immune control, including neutralising antibody and CD4^+^ and CD8^+^ T cells. Like HSV-2, HSV-1 can enter the stratified squamous oral and genital mucosa via the superficial epidermis, which consists mainly of keratinocytes, through minor abrasions. Keratinocytes are a formidable barrier to pathogen entry and form the keratinized superficial stratum corneum. Beneath this they are the major target for HSV infection, as they express nectin-1 which facilitates HSV entry. The stratum corneum is thin over the inner foreskin and labia and absent from the ectocervix, vagina and anus allowing easier viral entry into the epidermis [1,2].

The epidermis also contains the mononuclear phagocyte (MNP) population, Langerhans cells (LCs), which initiate the immune response to control primary HSV infections and limit local and axonal spread and colonization of the neuronal ganglia [3–5]. Therefore understanding the initial interaction of HSV with these cells is important. Until recently LCs were thought to be the sole MNP in uninflamed human epidermis. LCs perform a sentinel function, detecting and taking up pathogens, after which they usually mature and migrate to lymph nodes where they present their antigens to naïve T cells, thereby activating the adaptive immune response [6]. However murine HSV studies have shown that while LCs are important in the initial uptake of HSV [3], they do not present HSV antigen to naïve CD8^+^ T cells in lymph nodes. Rather this is the function of CD8α^+^ dendritic cells (MNPs) and CD103^+^ dermal MNPs (cDC1s) [7–9], which transport HSV antigens out of murine skin explants [10], suggesting that an exchange of HSV antigen between different MNP subtypes occurs in skin.

The role and response of human MNPs to HSV infection was first studied using model monocyte-derived MNPs (MDDCs) because of technical limitations in obtaining human skin and mucosal MNPs. These immature MDDCs could be productively infected by HSV, resulting in apoptosis. Apoptosis is a form of programmed cell death that occurs in a rapid and controlled manner, to avoid cell leakage and inflammation [11,12]. Virus infection can trigger the intrinsic apoptosis pathway, defined by the release of cytochrome c from the mitochondria, induction of the initiator caspase, caspase-9, followed by downstream activation of the executioner caspases-3, -6 and -7, leading to cell death [13,14] and clearance by phagocytes [11]. HSV is characteristically anti-apoptotic in Vero, Jurkat, epithelial and neuronal cells, with several viral anti-apoptotic mediators identified including ICP27, Us3, Us5, ICP4 and ICP10 [15–22]. Conversely, in myeloid cells, especially human and murine MDDCs and LCs, HSV infection induces apoptosis, via its ‘early protein’ expression and downregulation of c-FLIP and HSV latency-associated transcript (LAT), which are inhibitors of apoptosis [10,14,23–25]. Bystander uninfected MNPs pulsed with these apoptotic HSV-infected MDDCs could take them up and cross-present HSV antigens to and stimulate HSV specific CD8^+^ T cells [23,26]. This suggested a mechanism for viral antigen exchange between MNPs in the dermis.

Using topical HSV infection, our previous human foreskin explant studies have confirmed that LCs were shown to be productively infected by HSV and migrate into the dermis while undergoing apoptosis [27]. The migrating apoptotic HSV-1 infected LCs interacted with different MNP subsets in the dermis. It is now known that in human dermis, the two main DC subsets are conventional DC type 1 and 2 (cDC1 and cDC2) [28]. cDC1s are less frequent but are highly efficient at cross-presentation of endogenous antigen to CD8^+^ T cells [29]. cDC2s have conventional antigen-presenting capacity to stimulate CD4^+^ T cells, but also have some ability to cross-present to CD8^+^ T cells [30,31]. We showed that HSV-1 infected apoptotic LCs interacted with CD141^+^ cDC1s in clusters in the dermis. LC fragments were detected within some cDC1s, and cDC1s emigrated from HSV-1 infected explants, similar to cDC1 in murine models. Additionally, DC-SIGN^+^ MNPs, now thought to be monocyte-derived macrophages in uninflamed tissue [32,33], were also observed in clusters interacting with HSV-1 infected LCs in the dermis [27]. These observations were confirmed in biopsies of genital herpes. Therefore, epidermal LCs take up HSV, become infected and transfer the virus or viral antigens to subsets of dermal MNPs/macrophages, facilitating viral relay, probably leading to stimulation of CD4^+^ and CD8^+^ T cells in lymph nodes by different pathways.

Ongoing work in our lab has enabled extraction of skin MNPs in an immature state, enabling us to study how they interact with viruses [34]. We recently reported a novel DC located in the epidermis which expresses high levels of CD11c and CD1c and is phenotypically and transcriptomically very similar to dermal cDC2. They are abundant and predominate over LCs in a wide range of genital tissues including vagina, anus and foreskin. Compared to both LCs and dermal cDC2s, they produced more inflammatory cytokines, had a greater propensity to activate T cells and supported higher levels of HIV infection. After topical HIV infection of foreskin explants the virus was observed to interact with both types of epidermal MNPs [35]. We therefore wanted to investigate how HSV interacted with human epidermal cDC2s (Epi-cDC2) and LCs *ex vivo*.

HSV has been shown to enter host cells via different pathways, the predominant one depending on the host cell type, although multiple pathways may occur in the same cell. Epithelial cells such as keratinocytes, HeLa cells and Chinese hamster ovary (CHO)-nectin 1 cells have been shown to be infected by a low pH-dependent endocytic pathway [36,37]. In contrast, Vero and neuronal cells are infected by pH-independent fusion at the plasma membrane [36,37]. Alternatively, C10 mouse melanoma cells are infected via a pH-independent, rapid endocytic pathway [38]. The HSV entry pathways into human MNPs have not been previously identified. However, a study of betaherpesvirus human cytomegalovirus (HCMV) entry into MDDCs demonstrated that HCMV entered via a pH-independent, but actin- and cholesterol-dependent “macropinocytosis-like” pathway [39]. Similarly, vaccinia virus which is of comparable size to HSV, enters MDDCs via pH-independent, actin- and cholesterol-dependent macropinocytosis [40].

Here, we show that both LCs and Epi-cDC2s interact with HSV-1 infected keratinocytes and contain HSV-1 after topical infection of human inner foreskin explants. Furthermore, *in vitro* Epi-cDC2s show increased HSV-1 replication and expression of immediate early, structural and late HSV-1 proteins than LCs. In investigating the mechanisms of uptake and infection of both cells we show that expression of the surface receptors, nectin-1 and HVEM are similar, as is the degree of initial uptake. However the route of uptake differs: for LCs it is principally via low pH-dependent endocytosis and involving binding to the surface receptor Langerin (CD207). In contrast, uptake into Epi-cDC2s is pH-independent and does not rely on langerin. However, actin and cholesterol inhibitors do block HSV-1 entry, suggesting pH-independent endocytosis or macropinocytosis is the likely route of uptake. HSV induces apoptosis in both mononuclear phagocytes (MNPs), suggesting both may play a role in a relay of HSV (antigen) to dermal MNPs and subsequent presentation to T cells in draining lymph nodes.

## Results

### LCs and Epi-cDC2s interact with HSV-1 in the epidermis of inner foreskin explants

**Fig 1:**
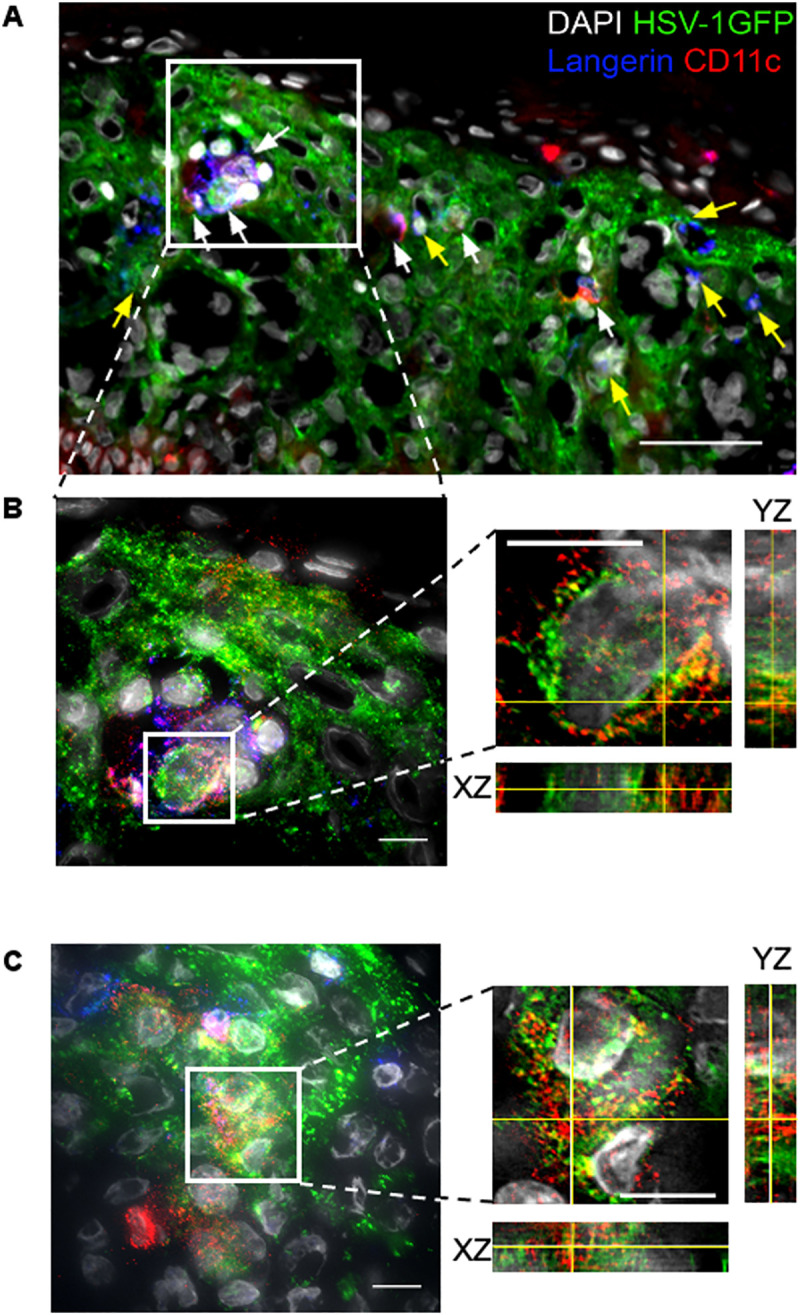
Interaction of LCs and Epi-cDC2s with HSV-1 in the epidermis of inner foreskin explants. Inner foreskin explants were topically infected with HSV-1 GFP or mock infected for 24 h, snap-frozen and cryo-sectioned prior to immunofluorescent labelling with anti-Langerin (blue) and anti-CD11c (red) antibodies and DAPI staining. **(A)** Image from a 9 y.o. donor shows LCs (Langerin^+^ CD11c^-^, blue cells) and Epi-cDC2s (CD11c^+^ Langerin^+/-^, red and red/blue dual labelled cells) in the epidermis of an HSV-1 GFP infected (green) foreskin explant at 20x magnification. Arrows indicate Epi-cDC2s (white arrows) and LCs (yellow arrows) interacting with HSV-1 GFP infected keratinocytes or that appear to be HSV-1 GFP^+^. This image is representative of the observations in three donors. Scale bar = 50 μm. **(B)** The same representative donor re-imaged at 60x magnification to generate Z-stacks with 0.1 μm Z spacing. The left panel shows the maximum intensity projection of 49 slices of a region of interest, indicated by the white box in (A). The right panel shows the orthogonal view of the XZ and YZ planes of an individual CD11c^+^ cell containing HSV-1 GFP^+^ labelling. Scale bar = 10 μm. **(C)** A 5 y.o. donor imaged as in (B) with the maximum intensity projection of 50 slices shown on the left and the orthogonal view of a CD11c^+^ cell containing HSV-1 GFP^+^ labelling on the right. Scale bar = 10 μm.

Previously, we showed that LCs become infected with HSV-1, and more recently that a second subset of MNPs resides in the epidermis, CD11c^+^ Epi-cDC2s. Firstly, we determined whether Epi-cDC2s also interact with HSV-1 *in situ*, in the epidermis of HSV-1 infected inner foreskin explants, using an HSV-1 strain F tagged with GFP at the US9 region (HSV-1 GFP). We observed that at 24 h, Epi-cDC2s (CD11c^+^ Langerin^+/-^) and LCs (Langerin^+^ CD11c^-^) both interacted with HSV-1 GFP infected keratinocytes (Fig 1A, indicated by white and yellow arrows respectively in a representative donor). In particular, Epi-cDC2s (red, CD11c^+^) appeared to contain HSV-1 GFP (green) in the cytoplasm (Fig 1B and 1C, images from two donors shown), indicative of uptake and probable infection of this second epidermal MNP subtype. Additional images confirming the presence of Epi-cDC2s and LCs in HSV-1 GFP^+^ epidermis, including a third donor are shown in S1 Fig.

### Epi-cDC2s show significantly greater HSV-1 replication than LCs *in vitro*

**Fig 2:**
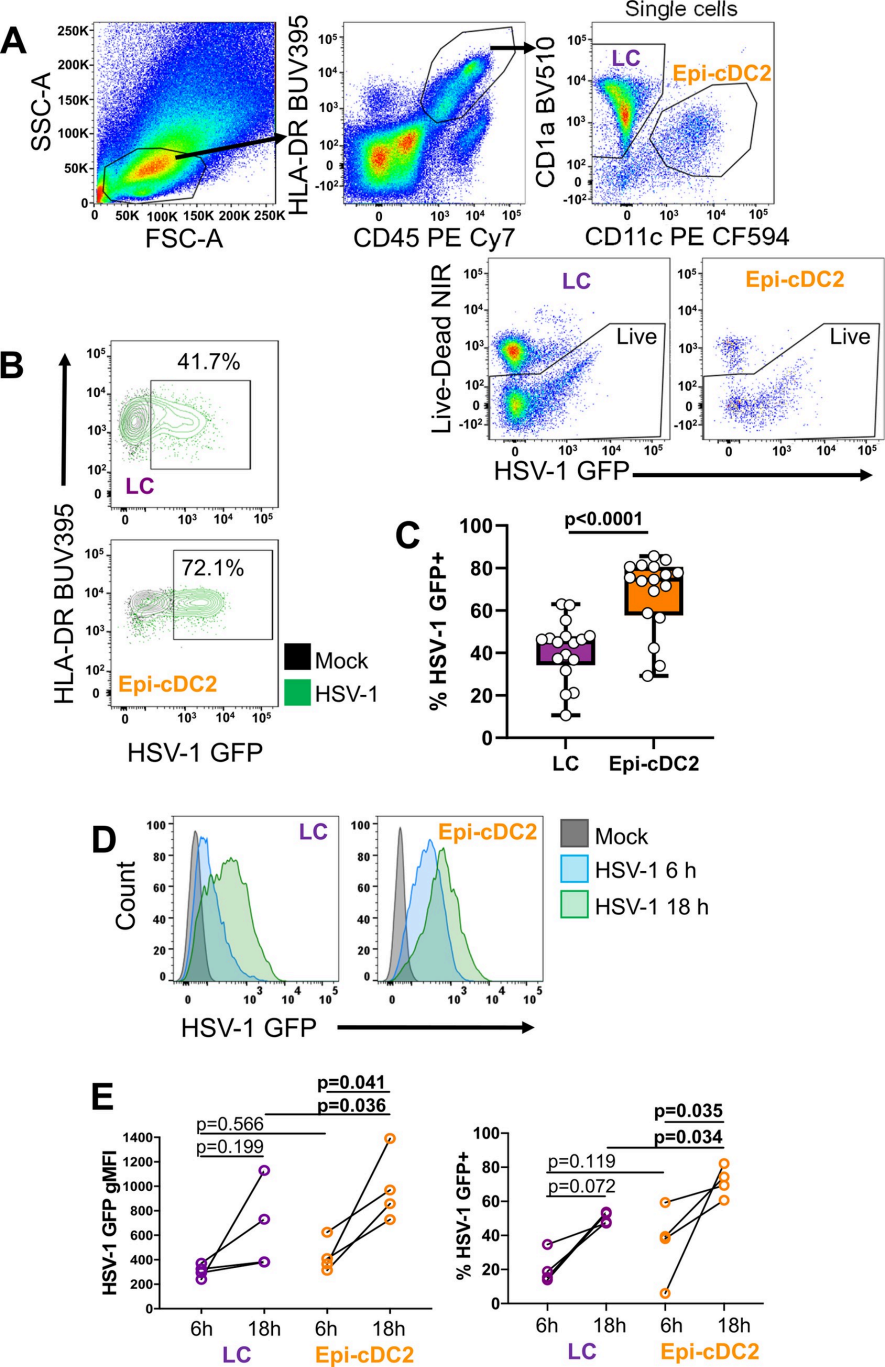
Epi-cDC2s are more extensively infected with HSV-1 than LCs. A mixed population of epidermal MNPs was isolated from human abdominal epidermis and inoculated with HSV-1 GFP (MOI of 10) or mock treated for 1 h, then washed and incubated for a total infection time of 18 h **(B & C)** or 6 and 18 h **(D & E)** in HaCaT-conditioned medium at 37°C. The cells were then washed with PBS and labelled with fixable LIVE/DEAD Near-IR followed by antibodies to HLA-DR, CD45, CD1a, CD11c, and Langerin, then analysed by flow cytometry. HSV-1 infection was detected using GFP expression. **(A)** Representative gating strategy to distinguish LCs and Epi-cDC2s. CD45^+^HLA-DR^+^ cells were gated on and separated into CD1a^+^CD11c^-^ LCs and CD1a^low^CD11c^+^ Epi-cDC2s. Live cells from each subset were then assessed for HSV infection by GFP expression for which a representative donor is shown in **(B). (C)** The percentage of HSV-1 GFP^+^ cells at 18 h in LCs (purple) and Epi-cDC2s (orange) is shown in box and whisker plots, where the central bar is the median, the box limits represent the upper and lower quartiles, the whiskers are the minimum and maximum values, and each dot represents an individual donor. A two-tailed paired t-test was used to compare the percentage of HSV-1 GFP^+^ LCs and Epi-cDC2s, n = 17**. (D)** Representative histograms of GFP expression at 6 and 18 h in LCs and Epi-cDC2s from one donor. **(E)** The expression of HSV-1 GFP, shown as gMFI, and the percentage of HSV-1 GFP^+^ cells for LCs (purple dots) and Epi-cDC2s (orange dots) at 6 and 18 h, time points are paired by donor. Repeated measures two-way ANOVA with Bonferroni’s test for pairwise comparisons were used to compare the gMFI or the % HSV-1 GFP^+^ LCs and Epi-cDC2s at 6 vs. 18 h, and LCs vs. Epi-cDC2s at each time point, n = 4.

Since we observed the interactions of both LCs and Epi-cDC2s with HSV-1 *in situ*, we conducted experiments using *ex vivo* isolated cells to quantify and compare the levels of HSV-1 infection in Epi-cDC2s with that of LCs. In previous work, we identified LCs using CD1a and langerin, which does not always discriminate between LCs and Epi-cDC2s. Here, we incorporated CD11c into our staining panel to reliably discriminate both cell types. Mixed epidermal MNPs (containing LCs and Epi-cDC2s) were isolated from healthy human abdominal skin specimens. To enhance the viability of the LCs, the cells were cultured in “HaCaT-conditioned medium”, a supplemented AIM V medium applied to a HaCaT monolayer (a spontaneously immortalised keratinocyte cell line) for 2 days, which was then harvested and used as the culturing medium for epidermal MNPs. The epidermal MNPs were infected with HSV-1 GFP for 18 h, sufficient time to allow for one round of HSV-1 replication. The proportion of HSV-1 GFP^+^ cells was then analysed in each cell type by flow cytometry (Fig 2A). After 18 h, both epidermal MNP types were infected with HSV-1 GFP and the proportion of HSV-1 GFP^+^ cells was significantly greater in Epi-cDC2s (67.7 ± 17.5%) compared to LCs (41.3 ± 14.2%) (Fig 2B and 2C). We also assessed the kinetics of HSV-1 GFP expression between 6 and 18 h. HSV-1 GFP expression, measured as geometric mean fluorescence intensity (gMFI) increased between 6 and 18 h in both LCs and Epi-cDC2s (Fig 2D and 2E left panel), indicative of increased virus expression. Additionally, at 18 h HSV-1 GFP expression (gMFI) was significantly greater in Epi-cDC2s than in LCs, whereas at 6 h there was no significant difference in HSV-1 GFP expression (Fig 2E, left panel). The proportion of HSV-1 GFP^+^ LCs and Epi-cDC2s was also assessed at 6 and 18 h and the results corresponded with the gMFI expression. At 6 h, a similar proportion of LCs and Epi-cDC2s were HSV-1 GFP^+^ (20.7 ± 9.5% and 35.7 ± 22.1% respectively) (Fig 2E, right panel). By 18 h an increased proportion of both LCs and Epi-cDC2s were HSV-1 GFP^+^ (50.4 ± 3.3% and 71.6 ± 9.0% respectively), suggestive of viral replication.

**Fig 3:**
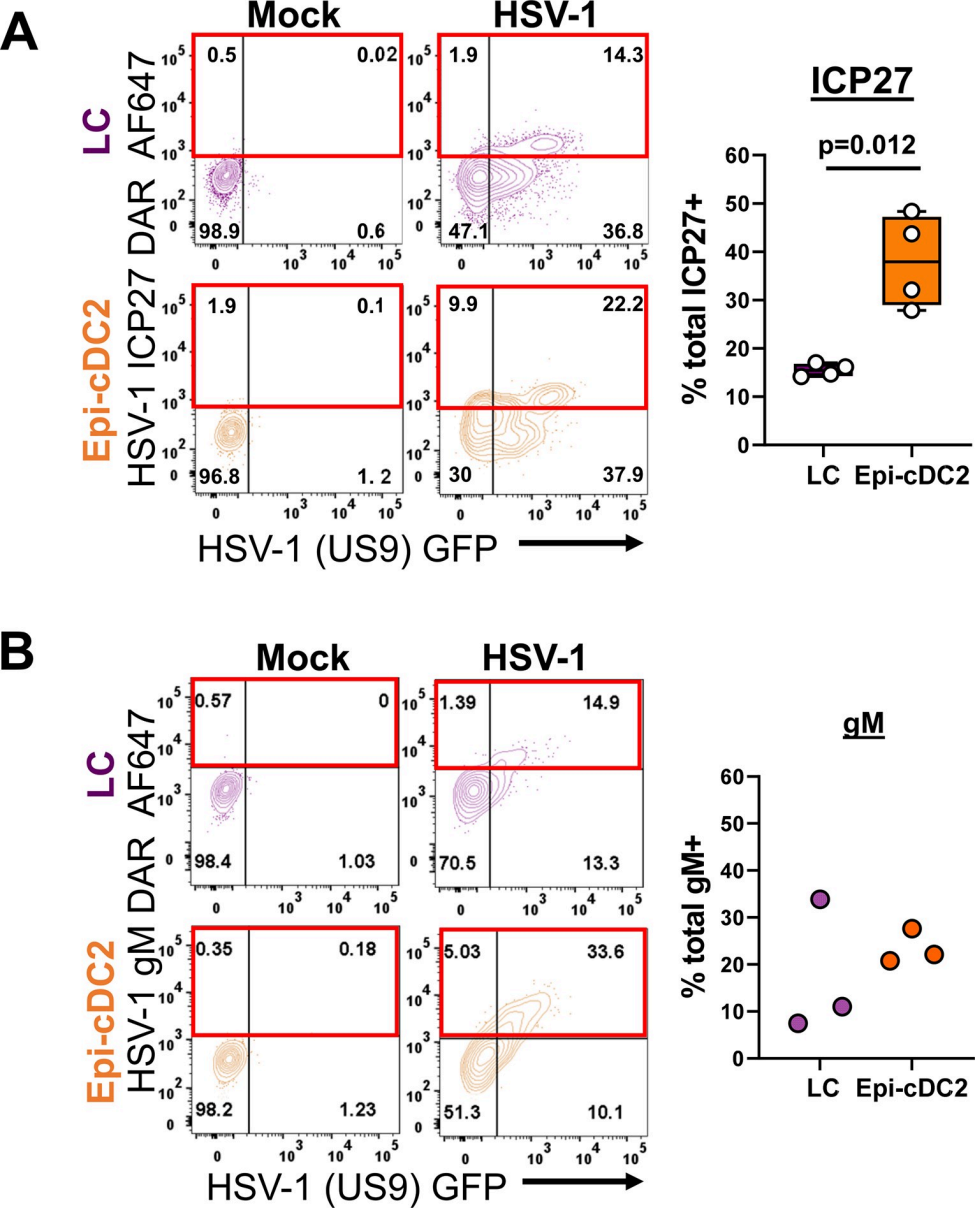
LCs and Epi-cDC2s express ICP27 and DNA-replication dependent late protein gM. A mixed population of epidermal MNPs was isolated from human abdominal epidermis and inoculated with HSV-1 GFP (MOI of 10) or mock treated for 1 h, then washed and incubated for a total infection time of 18 h in HaCaT-conditioned medium at 37°C. The cells were then washed with PBS and labelled for flow cytometry as in Fig 2. Additionally, cells were permeabilized then followed by serial intracellular labelling with **(A)** biotin-conjugated anti-ICP27 then Alexa Fluor-647 (AF647)-conjugated streptavidin, or **(B)** rabbit anti-HSV-1 gM then donkey-anti-rabbit (DAR) AF647. **(A)** The percentage of ICP27 AF647^+^ cells was analysed for each subset at 18 h, with gates set according to the mock condition. The graph shows box and whisker plots of the percentage of LCs (purple) and Epi-cDC2s (orange) expressing total ICP27, a combination of the ICP27^+^GFP^-^ and ICP27^+^GFP^+^ quadrants, indicated by the red boxes. A two-tailed paired t-test was used to compare the percentage of ICP27^+^ LCs and Epi-cDC2s, n = 4. **(B)** The percentage of gM AF647^+^ cells was analysed for each subset at 18 h, with gates set according to the mock condition. The graph shows the percentage of LCs (purple) and Epi-cDC2s (orange) expressing total gM, a combination of the gM^+^GFP^-^ and gM^+^GFP^+^ quadrants, indicated by the red boxes, n = 3.

**Fig 4:**
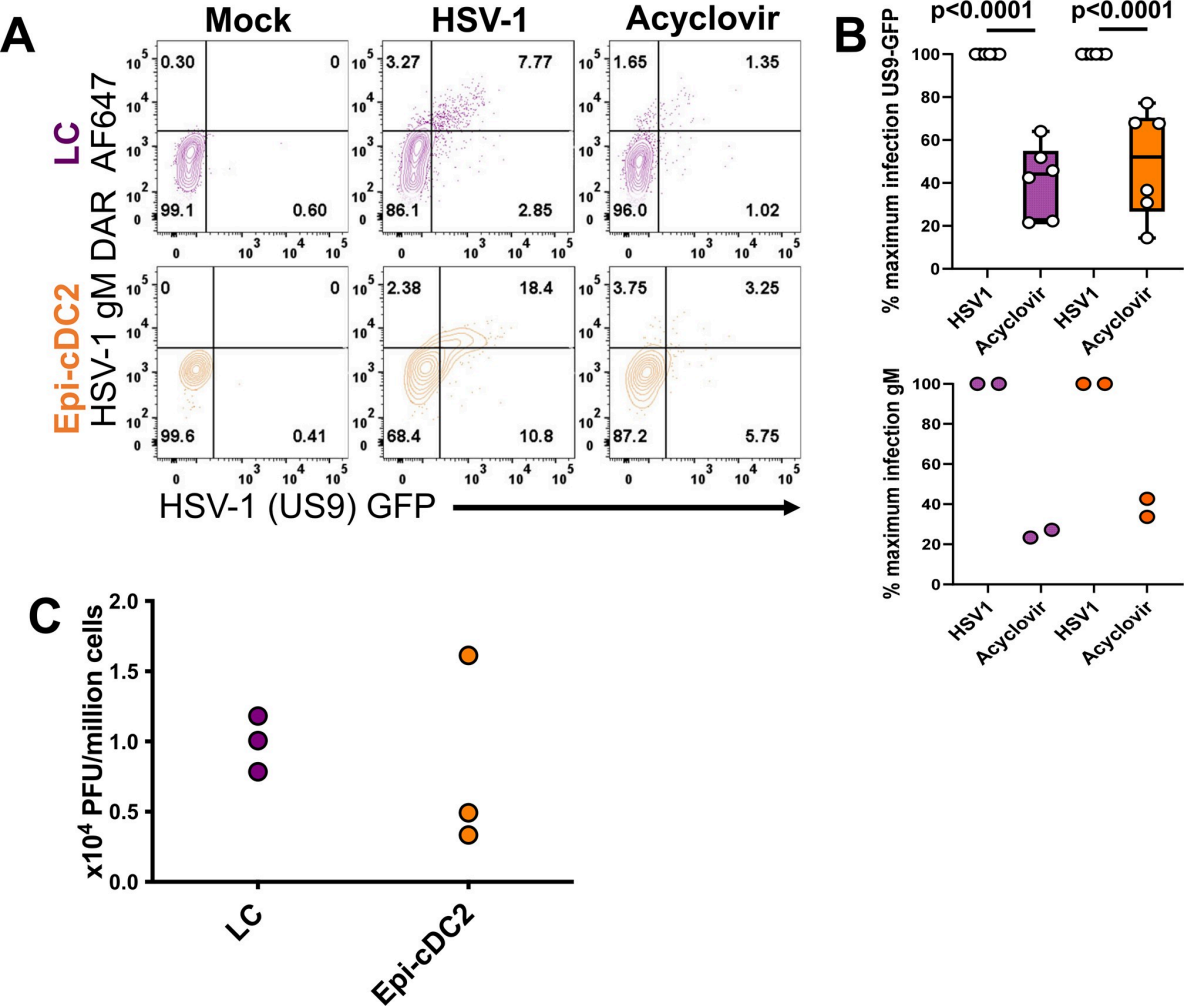
The expression of HSV-1(US9)-GFP and gM are significantly reduced by acyclovir, low level extracellular virus is detectable from both LCs and Epi-cDC2s. **(A & B)** A mixed population of epidermal MNPs was isolated from human abdominal epidermis and inoculated with HSV-1 GFP (MOI of 10) or mock treated for 1 h, then washed and resuspended in HaCaT-conditioned medium. HSV-1 GFP inoculated cells were split into two tubes, acyclovir (500 μg/ mL) was added to one tube and all cells were incubated for a total infection time of 18 h at 37°C. The cells were then washed with PBS and labelled for flow cytometry as in Fig 2. Additionally, cells were permeabilized then followed by serial intracellular labelling with rabbit anti-HSV-1 gM then donkey-anti-rabbit AF647. The percentage of HSV-1 (US9)-GFP^+^ and gM AF647^+^ cells was analysed for each subset at 18 h, with gates set according to the mock condition as shown in the **(A)** representative plot. **(B)** HSV-1 infected cells were set as 100% infection and the data are shown as box and whisker plots of the percentage of maximum infection in LCs (purple) and Epi-cDC2s (orange). Repeated measures one-way ANOVA with Tukey’s test for multiple pairwise comparisons was used to compare the percentage of maximum infection of each subset in the presence of acyclovir, HSV-1 (US9)-GFP n = 6, gM n = 2. **(C)** Mixed epidermal MNPs were isolated from human abdominal epidermis and FACS sorting was performed to isolate each subset. LCs and Epi-cDC2s were separately inoculated with HSV-1 GFP (MOI of 10) for 1 h, then washed, resuspended at 7x10^3^ cells per 100 μL and incubated for a total infection time of 24 h in HaCaT-conditioned medium at 37°C. Supernatants were collected and a fluorescent plaque assay was performed. Data shows the plaque counts of supernatants of HSV-1 infected LCs (purple) and Epi-cDC2s (orange) with PFU normalized to per million cells, n = 3.

To determine that HSV-1 replicates in Epi-cDC2s as we previously showed in LCs [27], expression of the nonstructural immediate early protein ICP27, an indicator of viral replication rather than uptake, was assessed at 18 h in both cell types, and a significantly greater proportion of Epi-cDC2s were ICP27^+^ than LCs (38.0 ± 9.6% and 15.5 ± 1.3% respectively) (Fig 3A). The GFP^+^ ICP27^-^ cells reflect either a decay in ‘early’ ICP27 production or higher sensitivity of GFP detection. Additionally we confirmed the expression of the strictly DNA replication-dependent HSV late protein glycoprotein M (gM) [41] in both Epi-cDC2s and LCs (23.5 ± 3.6% and 17.5 ± 14.3% respectively, Fig 3B), indicating the presence of immediate early, structural and true late protein expression is occurring within these cells. We then showed that the expression of HSV-1-(US9)-GFP and gM at 18 h were markedly and significantly reduced by acyclovir in both Epi-cDC2s and LCs with a significant reduction for HSV-1-(US9)-GFP (49.13 ± 25.3% and 41.3± 13.7% respectively compared to HSV-1 infection set to 100%) and a larger reduction observed in two donors for gM (Fig 4A and 4B). Extracellular production of infectious HSV by LCs and Epi-cDC2s was examined by plaque assay at 24 h and shown to have detectable (0.9 ± 0.19 x10^4^ PFU/million LCs, 0.8 ± 0.69 x10^4^ PFU/million Epi-cDC2s, Fig 4C) but on average 20–30 times lower output compared to supernatant derived from HSV-1 infected MDDCs (2x10^5^ PFU/million cells, [26]), consistent with partial inhibition of viral egress and/or assembly by the induction of apoptosis (discussed later). These results show that HSV replicates in both MNP subtypes and that by 18 h Epi-cDC2s were more extensively infected than LCs, even though extracellular infectious HSV production was very low in both cell types at 24 h.

### LCs and Epi-cDC2s both express HSV entry receptors HVEM and nectin-1

**Fig 5:**
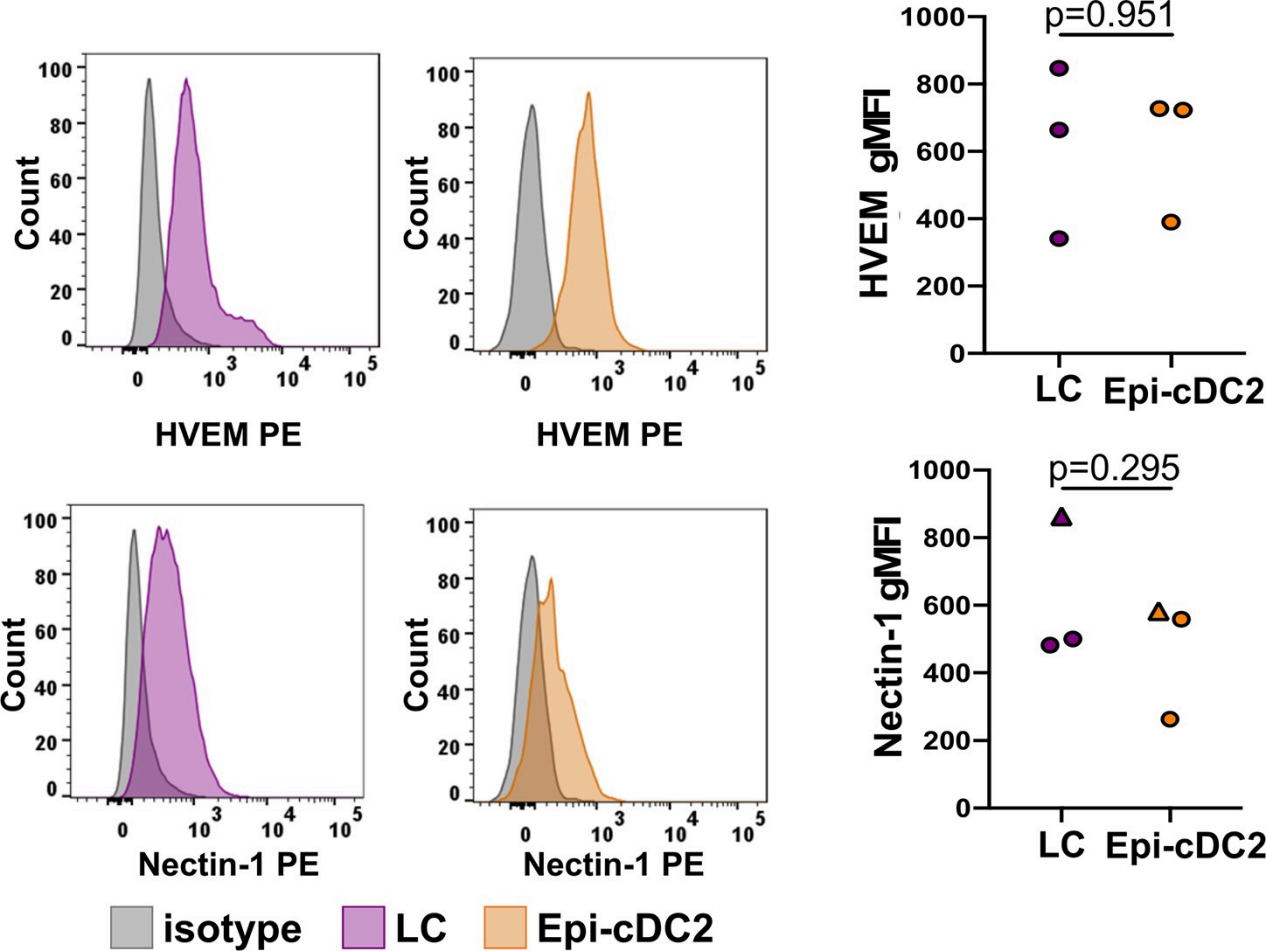
LCs and Epi-cDC2s both express HSV-1 entry receptors HVEM and nectin-1. Epidermal MNPs were isolated from human abdominal or foreskin epidermis and labelled for flow cytometry as in Fig 2, as well as with antibodies to HVEM or nectin-1. The surface expression of HVEM and nectin-1 on LCs (purple histograms) and Epi-cDC2s (orange histograms) is shown in a representative donor. Mean receptor expression (gMFI) is graphed for LCs (purple) and Epi-cDC2s (orange). Circles indicate abdominal skin, triangles indicate foreskin. Two-tailed paired t-tests were used to compare the gMFI of HVEM or nectin-1 expression in LC and Epi-cDC2s, n = 3.

As we observed marked increases in the level of HSV infection in Epi-cDC2s compared to LCs, we next compared their expression of the HSV entry receptors HVEM and nectin-1 as a first step in comparing virus uptake between the two cell types. We observed no significant difference in the levels of HVEM and nectin-1 expression in Epi-cDC2s compared to LCs and no significant difference in expression levels between cells derived from abdominal skin and foreskin (Fig 5). This was consistent with the observation in the previous result that at 6 h there was no significant difference in the expression of HSV-1 GFP in LCs and Epi-cDC2s, indicating a similar level of initial uptake (Fig 2E).

### HSV-1 entry into MDDCs is low pH-dependent, like HeLa cells

**Fig 6:**
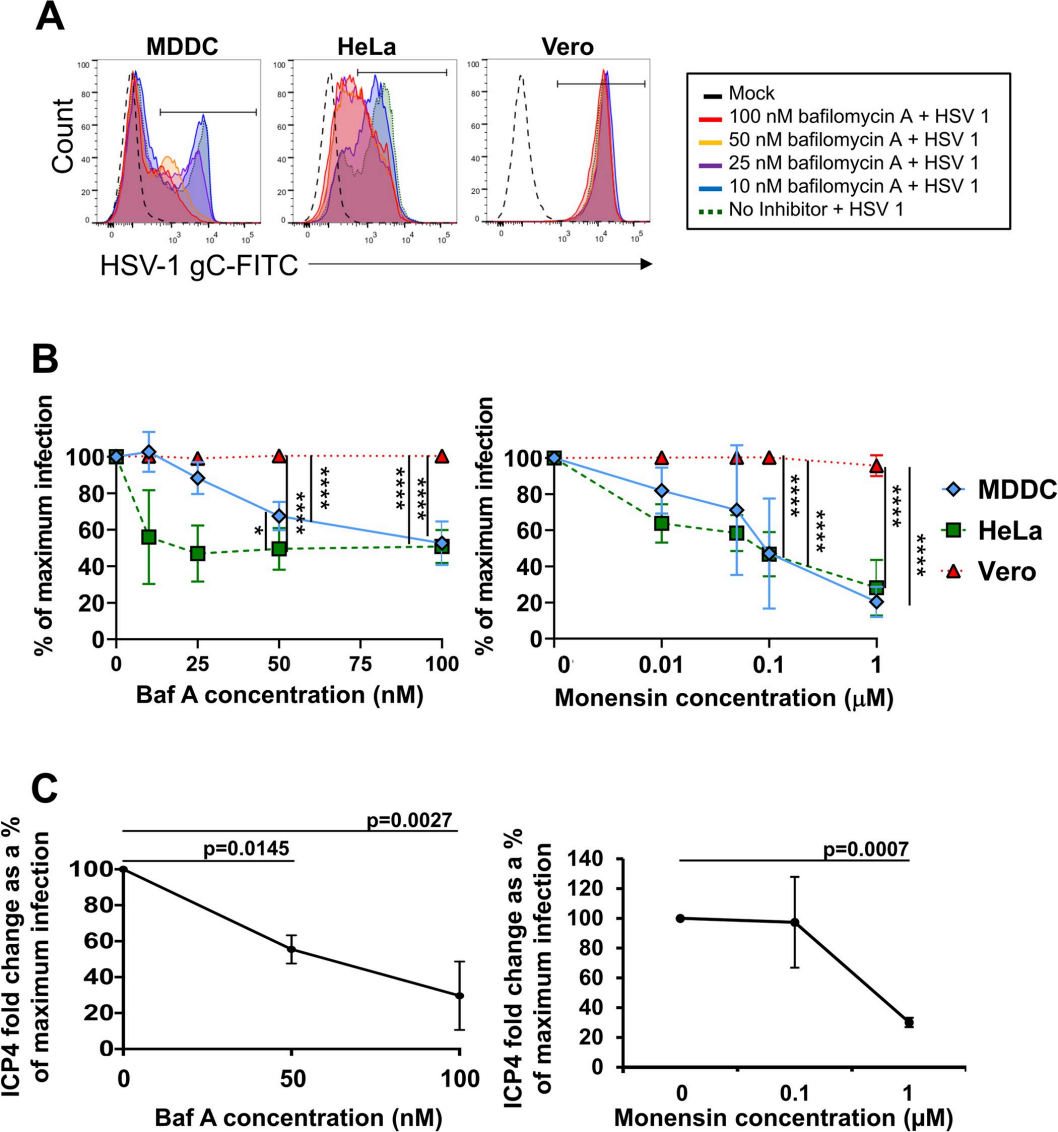
HSV-1 entry into MDDCs is pH-dependent and endocytic. MDDCs, HeLa and Vero cells were pre-treated with bafilomycin A (baf A) or monensin for 30 min at 37°C at the concentrations shown. HSV-1 (strain F) was added (MOI of 3) in the continued presence of the agents and allowed to bind for 1 h at 37°C. Cells were then washed and incubated in the presence of inhibitors at 37°C for a total infection time of 18 h. **(A** and **B)** Cells were labelled with anti-HSV-1 gC antibody (HSV-1 gC-FITC), then analysed by flow cytometry. **(A)** Representative histograms of gC expression in each cell type. **(B)** Percentage of HSV-1 gC-FITC^+^ cells with increasing concentrations of baf A or monensin for each of the three cell types at 18 h. HSV-1 infected cells with no inhibitor were set as 100% infection and the data are shown as the percentage of maximum infection. Two-way ANOVA with Tukey’s test for multiple pairwise comparisons was used to compare the percentage of maximum infection between cell types at each concentration of each inhibitor, n = 4 (HeLa n = 3). The ANOVA summary p values for variation in inhibitor concentration and cell type were p<0.0001 for both inhibitors. Significant pairwise p values: *50 nM baf A*: HeLa vs. Vero p<0.0001, MDDC vs. Vero p<0.0001, MDDC vs. HeLa p = 0.0390, *100 nM baf A*: HeLa vs. Vero p<0.0001, MDDC vs. Vero p<0.0001, *0*.*1 μM monensin*: HeLa vs. Vero p<0.0001, MDDC vs. Vero p<0.0001, *1 μM monensin*: HeLa vs. Vero p<0.0001, MDDC vs. Vero p<0.0001. **(C)** Following infection, MDDCs were washed, lysates were collected, and qPCR was performed to detect mRNA for the HSV-1 immediate early gene ICP4. Fold change relative to mock was calculated, the no inhibitor HSV-1 infected MDDCs were set as 100% infection and the data shows ICP4 expression as a percentage of maximum infection in baf A (left panel) or monensin (right panel) treated MDDCs. Repeated measures one-way ANOVA with Bonferroni’s test for pairwise comparisons were used to compare the percentage of maximum infection of each concentration of baf A or monensin to the no inhibitor (0 nM/μM) control, n = 3.

We next investigated the pathway of HSV-1 entry into each epidermal MNP type. As *in vitro* differentiated MDDCs are known to be infected by HSV-1, and have been a convenient and widely used model for viral infection of MNPs [23,26,42–48], we first investigated which entry pathway was utilised by HSV-1 into MDDCs. As previously published, Vero and HeLa cells are infected by HSV-1 via different entry pathways. HSV-1 enters HeLa cells via low pH-dependent endocytosis which can be blocked by lysosomotropic agents such as the vacuolar ATPases inhibitor bafilomycin A, whereas HSV-1 enters Vero cells via neutral fusion and is not affected by bafilomycin A [37]. Therefore, to determine whether HSV-1 entry into MDDCs was pH-dependent, MDDCs, and as controls Vero and HeLa cells, were pre-incubated with bafilomycin A, then infected with HSV-1 for 18 h in the presence of the inhibitor. Pilot studies in MDDCs showed no increase in cell toxicity at concentrations of bafilomycin A of 10–100 nM (S2A Fig). A significant dose-dependent reduction in the expression of HSV-1 glycoprotein C (gC) was observed in both HeLa cells and MDDCs but not Vero cells. Compared to no inhibitor controls (set to 100%), at the highest concentration of bafilomycin A (100 nM), infection of both MDDCs and HeLa cells was significantly reduced (to 52.5 ± 11.9% and 50.8 ± 9% respectively), while infection of Vero cells was unaffected as expected (Fig 6A and 6B, left panel). Similarly, the effect of a different lysosomotropic agent, the protein transport inhibitor monensin was also examined and a significant dose-dependent inhibition of HSV infection was also observed. At the highest concentration (1 μM), monensin also significantly reduced HSV-1 infection in MDDCs and HeLa cells (to 20 ± 8% and 28 ± 15% respectively), but not Vero cells (Fig 6B, right panel).

To determine whether bafilomycin A inhibited the earliest stages of HSV replication i.e. HSV-1 entry into MDDCs, immediate early gene ICP4 RNA was measured by qPCR. Bafilomycin A significantly reduced ICP4 RNA at both 50 and 100 nM concentrations compared to HSV-1 infected controls (no inhibitor; Fig 6C, left panel). This was also observed with 1 μM of monensin compared to HSV-1 infected controls (Fig 6C, right panel). Therefore, HSV-1 entry into MDDCs, like HeLa cells, was low pH-dependent.

### HSV-1 entry is low pH-dependent and langerin-dependent in LCs but not Epi-cDC2s

**Fig 7:**
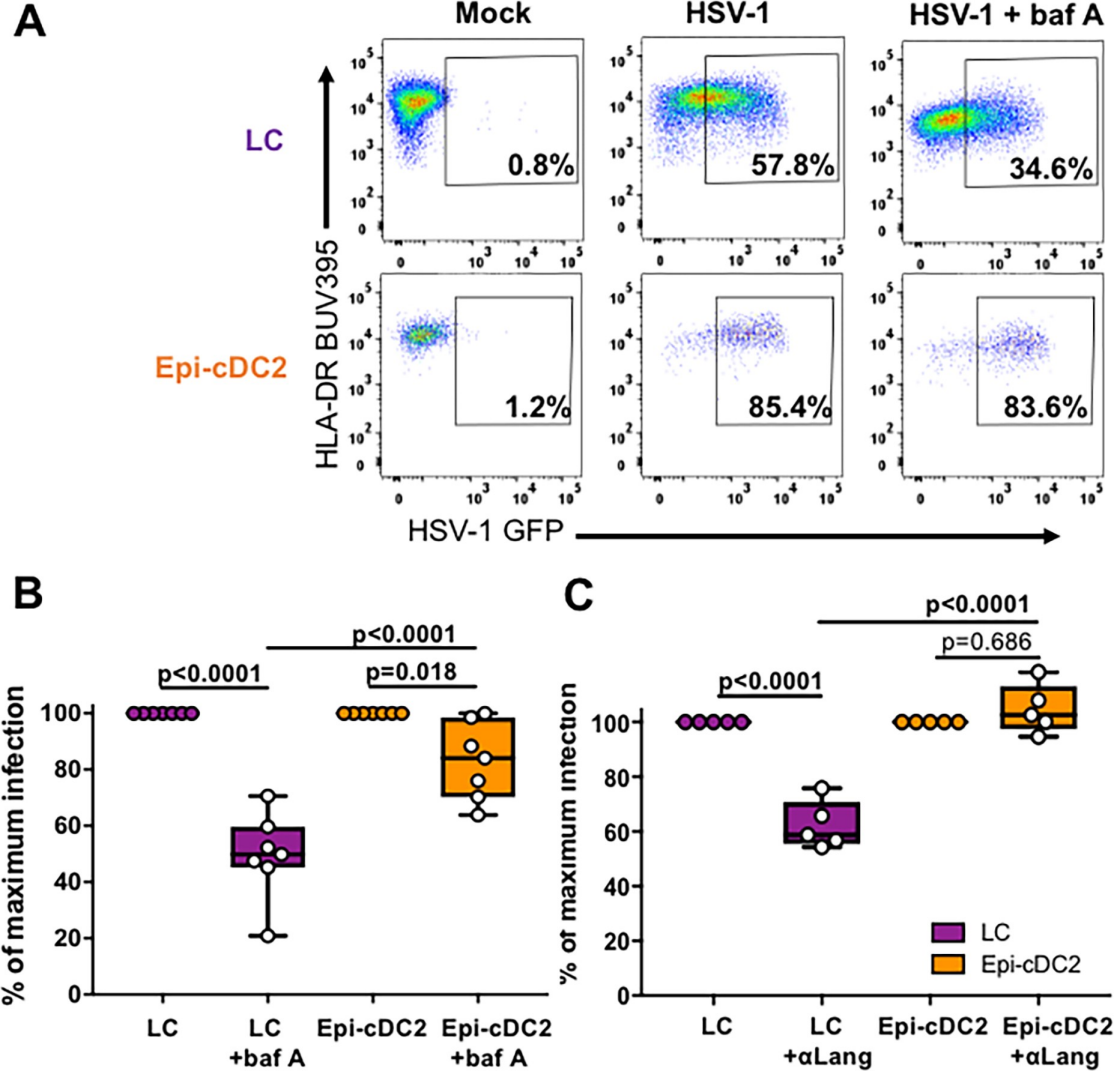
HSV-1 entry into LCs is pH-dependent and langerin-dependent. Isolated abdominal epidermal MNPs were pre-treated with 100 nM bafilomycin A (baf A) for 30 min at 37°C **(A** and **B)** or with 5 μg/mL anti-langerin antibody **(C)** then infected with HSV-1 GFP for 18 h and stained for flow cytometry as in Fig 2, to detect infection using GFP expression. **(A)** Representative plots of HSV-1 infection in the absence or presence of baf A. The percentage of HSV-1 GFP^+^ cells was measured with and without **(B)** baf A (n = 7) or **(C)** anti-langerin antibody (n = 5) at 18 h. HSV-1 infected cells were set as 100% infection and the data are shown as box and whisker plots of the percentage of maximum infection in LCs (purple) and Epi-cDC2s (orange). Repeated measures one-way ANOVA with Tukey’s test for multiple pairwise comparisons was used to compare the percentage of maximum infection of each subset in the presence of inhibitor.

To investigate low pH-dependent entry into epidermal MNPs we concentrated on using bafilomycin A because it has specific effects in inhibiting endosomal acidification and because our data showed it had no additional toxic effects above the cell death seen in mock or HSV-1 infected cultures (S2B Fig). Epidermal MNPs were pre-incubated with 100 nM bafilomycin A (the concentration with the most significant inhibition in MDDCs), then infected for 18 h with HSV-1 GFP in the presence of the inhibitor. LCs had a marked and significant reduction in HSV-1 infection (to 49.4 ± 15.3%) in the presence of bafilomycin A compared to HSV-1 alone (set to 100%), whereas Epi-cDC2s had a minor reduction in infection (to 83.0 ± 13.8%) in the presence of bafilomycin A (Fig 7A and 7B). The infection of LCs in the presence of bafilomycin A was also significantly reduced compared to Epi-cDC2s. Therefore, HSV-1 entry into LCs was largely low pH-dependent, whereas entry into Epi-cDC2s was not.

We next investigated whether the C-type lectin receptor, langerin, is required for HSV-1 endocytic entry into LCs, as has been shown for HIV and Influenza A [49,50]. We compared HSV-1 infection of LCs and Epi-cDC2s in the presence of an anti-langerin neutralizing antibody, at two concentrations, and found that LCs had a significant reduction in infection (to 62.3 ± 8.7%) in the presence of the antibody, whereas for Epi-cDC2s no reduction in infection was observed (104.7 ± 8.9%), even though a small subset of the Epi-cDC2s expressed low levels of langerin (Fig 7C). Therefore, HSV-1 entry into LCs was mediated by langerin and a low pH-dependent endocytic pathway, whereas entry into Epi-cDC2s was not.

### HSV-1 entry into Epi-cDC2s is mediated by actin and cholesterol

**Fig 8:**
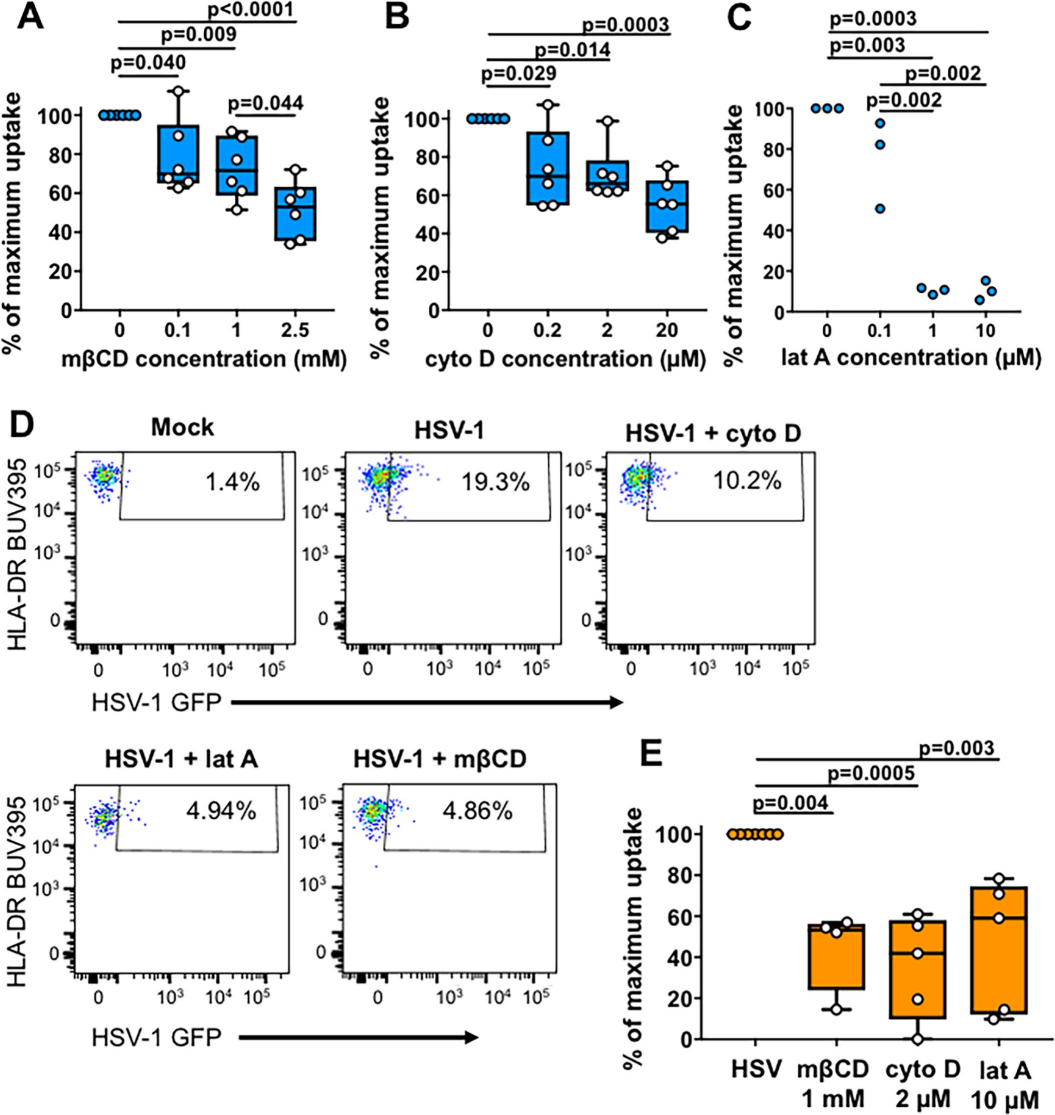
HSV-1 uptake into MDDCs and Epi-cDC2s is dependent on actin and cholesterol. MDDCs or isolated abdominal epidermal MNPs were pre-treated for 30 min at 37°C with mβCD, cytochalasin D (cyto D), latrunculin A (lat A) or no inhibitor at the concentrations shown. HSV-1 GFP was added (MOI of 3 or 10 for MDDCs and epidermal MNPs respectively) in the presence of the inhibitor and allowed to bind for 1 h at 4°C. The cells were washed and incubated for a further 1 h at 37°C in the continued presence of the inhibitor, then trypsinised for 15 min at 37°C to remove surface bound virus. Cells were labelled for flow cytometry as in Fig 2. **(A-C)** The percentage of HSV-1 GFP^+^ MDDCs was measured by flow cytometry for **(A)** mβCD (n = 6) **(B)** cyto D (n = 6) or **(C)** lat A (n = 3) treated conditions. HSV-1 infected cells with no inhibitor were set as 100% uptake and the data are shown as box and whisker plots of the percentage of maximum uptake. Repeated measures one-way ANOVA with Tukey’s test for multiple pairwise comparisons was used to compare the percentage of maximum uptake of MDDCs in the presence of each inhibitor. **(D)** Representative donors showing the percentage of HSV-1 GFP^+^ cells in the presence of cyto D, lat A or mβCD compared to HSV-1 only in Epi-cDC2s. **(E)** HSV-1 infected Epi-cDC2s with no inhibitor were set as 100% uptake and the data are shown as box and whisker plots of the percentage of maximum uptake. One-way ANOVA with Tukey’s test for multiple pairwise comparisons was used to compare the percentage of maximum uptake of Epi-cDC2s in the presence of each inhibitor. HSV n = 8, mβCD n = 4, cyto D n = 6, lat A n = 5.

We next focussed on Epi-cDC2s to investigate pH-independent HSV-1 uptake pathways using MDDCs as controls. These short uptake experiments rely on detecting GFP on the input virus which is lower than the amplified GFP signal seen in longer infection experiments (Figs 2, 3 and 6). We chose actin inhibitors cytochalasin D and latrunculin A and cholesterol inhibitor methyl-β-cyclodextrin (mβCD), which have been used in previous studies to demonstrate clathrin-independent endocytosis and macropinocytosis [39,40,51,52]. We first tested the dose-dependent effect of these inhibitors on MDDCs. MDDCs were pre-treated with each inhibitor prior to addition of HSV-1 GFP. Compared to HSV-1 only controls (set as 100%), a significant dose-dependent reduction in the percentage of HSV-1 GFP^+^ MDDCs was observed in MDDCs treated with mβCD (to 51.4 ± 14.7%) (Fig 8A). Similarly, significant dose-dependent reductions in the percentage of HSV-1 GFP^+^ MDDCs were also observed in those treated with cytochalasin D (to 55.1 ± 14.2%) and latrunculin A (to 10.4 ± 4.8%) (Fig 8B and 8C).

Following this, we next applied these inhibitors to epidermal MNPs prior to addition of HSV-1 GFP as described for the MDDCs. Compared to HSV-1 alone (set to 100%), Epi-cDC2s had donor variable but significant reductions in HSV-1 GFP^+^ cells in the presence of mβCD (to 44.5 ± 20.1%), cytochalasin D (to 35.6 ± 25.5%) and latrunculin A (to 46.5 ± 32.2%) (Fig 8D and 8E). Therefore, cholesterol and actin are important mediators of HSV-1 entry and subsequent replication in Epi-cDC2s.

### HSV induces apoptosis of infected and bystander LCs and Epi-cDC2s

**Fig 9:**
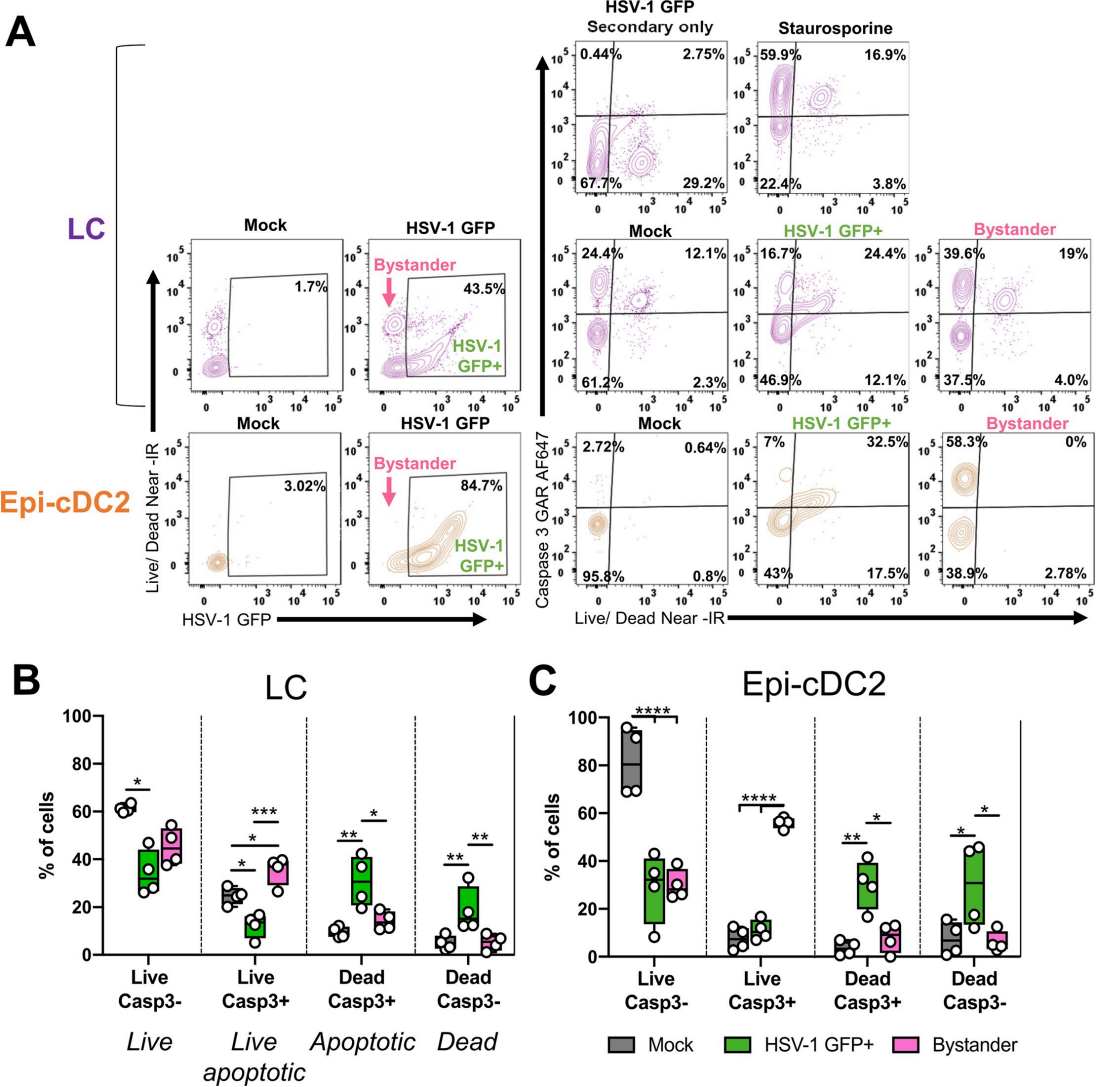
HSV-1 induces apoptosis of both infected and bystander LCs and Epi-cDC2s. Epidermal MNPs were isolated from human abdominal epidermis and inoculated with HSV-1 GFP (MOI of 10) or mock treated, then washed and incubated for a total infection time of 18 h. As a positive control, some cells were treated with 1 μM staurosporine for 18 h at 37°C. The cells were labelled for flow cytometry as in Fig 2, as well as with intracellular active Caspase-3 (Casp3) and LIVE/DEAD Near-IR for examining apoptosis. **(A)** Representative plots showing apoptosis analysis. In the HSV-1 infected conditions, LCs and Epi-cDC2s were first separated into *HSV-1 GFP+* and *Bystander* (HSV-1 GFP-) populations including live and dead cells. Apoptosis was assessed in mock, HSV-1 GFP and staurosporine treated conditions by analyzing the distribution of cells over the four Casp3 vs. Live/Dead quadrants, with gates set according to the mock and staurosporine conditions. Live Casp3-: *live cells*, Live Casp3+: *live apoptotic cells*, Dead Casp3+: *apoptotic cells*, Dead Casp3-: *dead cells*. **(B** and **C)** Grouped analyses of the percentage of LCs and Epi-cDC2s (respectively) in each of the apoptosis quadrants in mock (grey), HSV-1 GFP+ (green) and Bystander (pink) populations shown as box and whisker plots. Repeated measures one-way ANOVA with Tukey’s test for multiple pairwise comparisons was used to compare the percentage of LCs **(B)** or Epi-cDC2s **(C)** in each apoptosis quadrant between mock, HSV-1 GFP+ and bystander populations, n = 4. **(B)** *Live cells*: Mock vs. HSV-1+ p = 0.0100, *Live apoptotic cells*: Mock vs. HSV-1+ p = 0.0140, Mock vs. Bystander p = 0.0245, HSV-1+ vs. Bystander p = 0.0006, *Apoptotic cells*: Mock vs. HSV-1+ p = 0.0100, HSV-1+ vs. Bystander p = 0.0296, *Dead cells*: Mock vs. HSV-1+ p = 0.0064, HSV-1+ vs. Bystander p = 0.0070. **(C)** *Live cells*: Mock vs. HSV-1+ p<0.0001, Mock vs. Bystander p<0.0001, *Live apoptotic cells*: Mock vs. Bystander p<0.0001, HSV-1+ vs. Bystander p<0.0001, *Apoptotic cells*: Mock vs. HSV-1+ p = 0.0070, HSV-1+ vs. Bystander p = 0.0158, *Dead cells*: Mock vs. HSV-1+ p = 0.0172, HSV-1+ vs. Bystander p = 0.0133.

We previously showed that HSV induced apoptosis in MDDCs via its early proteins [23]. Furthermore HSV infected LCs that migrated to the dermis in foreskin explants underwent apoptosis, identified by shrunken and fragmented morphology and the expression of caspase-3 [27]. Since we observed here that both subtypes of epidermal MNPs were infected by HSV, we next assessed whether Epi-cDC2s, like LCs, undergo apoptosis following HSV infection. This was done by flow cytometry to gate separately on infected (HSV-1 GFP^+^) and bystander (HSV-1 GFP^-^) cells in the HSV infected cultures, and to differentiate stages of apoptosis into quadrants with the combination of caspase-3 and a viability dye: *live cells* (Live Casp3-), *live apoptotic cells* (Live Casp3+), *apoptotic cells* (Dead Casp3+) and *dead cells* (Dead Casp3-) (Fig 9A). In this experiment, LCs had lower viability, even in mock samples, than Epi-cDC2s as they are typically more fragile in culture than Epi-cDC2s.

After 18 h, a significant increase in apoptosis and cell death was observed in HSV-1 GFP^+^ cells in both MNP subtypes. As expected, when compared to the mock, HSV-1 GFP^+^ infected LCs had a higher proportion of *apoptotic cells* (30.8 ± 10.6% vs. 9.6 ± 2.3%) and *dead cells* (18.7 ± 9.5% vs. 4.9 ± 2.9%) while there was little change in the bystander populations (Fig 9B). We found that HSV-1 GFP^+^ infected Epi-cDC2s, compared to mock, also had significantly increased *apoptotic cells* (30.0 ± 10.3% vs. 3.6 ± 3.0%) and *dead cells* (29.9 ± 17.6% vs. 7.5 ± 6.9%) and again there was little change in the bystander populations (Fig 9C). Therefore HSV-1 infection does induce apoptosis in Epi-cDC2s, as in LCs. HSV-1 exposure also lead to an increase in *live apoptotic cells* (early apoptotic cells) in the bystander populations compared to mock, for both MNP subtypes (LCs: 35.4 ± 6.0% vs. 24.5 ± 3.5%; Epi-cDC2s: 56.0 ± 2.3% vs. 7.5 ± 4.7%) (Fig 9B and 9C). When we compared between the two MNP subtypes, amongst the HSV-1 GFP^+^ population there was no statistically significant difference between LCs and Epi-cDC2s in the proportion of cells in each apoptosis quadrant. However, the increase in *live apoptotic* bystander cells was significantly greater in Epi-cDC2s than in LCs (S3 Fig). This suggested that bystander Epi-cDC2s were apoptosing to a greater degree than bystander LCs, possibly due to higher levels of early stage infection than LCs, not yet visible as GFP expression.

Overall, both LCs and Epi-cDC2s underwent apoptosis following HSV-1 infection, with cells that were HSV1-GFP^+^ being mostly apoptotic or dead by 18 h, while bystander cells were mostly live although with a significant proportion of live apoptotic cells.

## Discussion

In addition to LCs, we have recently described a second mononuclear phagocyte (MNP) population in human epidermis, enriched in anogenital tissues, that is transcriptionally and phenotypically related to dermal cDC2s, referred to here as Epi-cDC2s [35]. The two MNPs are best differentiated by the expression of CD11c on Epi-cDC2s [35]. When GFP labelled HSV-1 was topically applied to inner foreskin explants, both types of epidermal MNPs were observed to interact with infected keratinocytes. In addition, GFP labelled HSV-1 colocalized with both types of MNP indicating uptake and probably infection, as indicated by the distribution through the cytoplasm. Both Epi-cDC2s and LCs expressed the true late protein gM, which has been shown to be dependent on DNA replication [41]. The Epi-cDC2s showed a higher degree of viral replication *in vitro* than LCs, as indicated by 1. the greater increase in viral structural US9-GFP protein expression over 18 hours, 2. its significant inhibition by acyclovir together with marked inhibition of late protein gM 3. greater expression of the nonstructural protein ICP27 also at this time point. However this viral replication was not reflected in the low levels of extracellular infectious virus for both LCs and Epi-cDC2s at 24 h, probably a reflection of inhibition of viral egress or assembly by apoptosis.

In dissecting the mechanism we showed initially that both MNP subtypes expressed similar levels of the HSV entry receptors, nectin-1 and HVEM and the degree of HSV uptake was similar at early time points. Currently, it is known that HSV utilizes at least three different entry pathways to infect different cell types, a pH-dependent endocytic pathway, a pH-independent endocytic pathway, and a pH-independent fusion pathway at the plasma membrane. Fusion at the plasma membrane occurs during HSV entry into neurones whereas HSV enters epithelial cells via pH and clathrin-dependent endocytosis. Using inhibitors of endosomal acidification, and actin assembly, by flow cytometry we have shown that HSV-1 predominantly utilises a pH-dependent pathway to infect LCs (and MDDCs) but Epi-cDC2s are mostly infected via a pH-independent uptake pathway, susceptible to inhibitors of cholesterol and actin assembly. This suggests pH-independent endocytosis or macropinocytosis may be the dominant uptake pathway in these MNPs. It is difficult to define the different mechanisms of endocytic uptake of any pathogen because of overlapping effects of some inhibitors on multiple pathways [40,52–55]. Macropinocytosis is highly dependent on actin rearrangement but so are other types of endocytosis, although to a lesser degree. However in LCs the specific effect of bafilomycin A on vacuolar ATPase and endocytic acidification showed low pH-dependent endocytosis was dominant in these cells, strongly suggesting the mechanism was clathrin-dependent. The lack of effect on epidermal cDC2s defines the difference in endocytic pathways between the two MNP subtypes. The combination of this lack of inhibition by bafilomycin A and inhibition by actin inhibitors strongly suggests macropinocytosis as the endocytic pathway in Epi-cDC2s. A possible confounder is inhibition of HSV transport through the cortical actin skeleton after entry in the latter, but this would not explain the lack of bafilomycin A inhibition of uptake. It is most interesting that entry into the two different types of epidermal MNPs utilizes these two different pathways of pH- and non-pH dependent endocytosis. It has been demonstrated that CHO cells engineered to express human nectin-1 are susceptible to infection via a pH-dependent route whereas CHO cells expressing human paired immunoglobulin-like type 2 receptor alpha (PILRα) are infected via a pH-independent route [56], possibly by associating with gB. As the receptor repertoire between LCs and cDC2s is vastly different [35,57], we hypothesise a receptor on cDC2s but not LCs may play the same role as PILRα does on CHO-PILRα cells. Additionally another recent study using CHO-HVEM cells has identified a nonconventional endocytic pathway of HSV-1 entry, independent of the involvement of Rab GTPases that are required in the canonical endosomal-lysosomal pathway [58]. Relative uptake into these two epidermal MNP cell types, with their differences in pH dependence, at superficial genital tract locations might also be influenced by the pH of the genital tract, especially in the vagina with its constitutive low pH, but which can be neutralized by semen [59–63].

As there is little difference in HSV receptor expression and early uptake between the two epidermal MNPs this suggests that the higher levels of productive infection of Epi-cDC2s may be at least partly due to different pathways of viral replication or viral degradation initiated by endocytic uptake pathways for LCs and Epi-cDC2s. These are currently being investigated. Many C-type lectin receptors, including langerin, facilitate endocytic uptake of viruses as shown here for LCs. We have previously shown langerin is expressed on Epi-cDC2s in genital mucosa but at lower levels on those from abdominal skin [35]. Further studies to examine HSV uptake in genital skin are required and also to define the other CLRs able to facilitate uptake of HSV in Epi-cDC2s.

HSV induced apoptosis in both epidermal MNP types as we previously reported for LCs and MDDCs [23,27]. However at 24 h, there was 20- to 30-fold less extracellular infectious HSV produced compared to MDDCs [26]. These differences probably reflect differences in the kinetics and functional effects of apoptosis in tissue LCs/MNPs when compared to blood MDDCs, reflecting the importance of studying authentic tissue cell types. The induction of apoptosis in these myeloids cells is in common, but contrasts with the multiple anti-apoptotic viral proteins and mechanisms induced during infection of epithelial cells and neurones [16,19–22]. Induction of apoptosis also inhibits direct antigen presentation to T cells but facilitates uptake by bystander MNPs, as we demonstrated in MDDC models and with epidermal LCs and dermal MNPs from abdominal skin. Therefore, it seems highly likely that the ‘HSV antigen relay’ between HSV infected apoptosing LCs migrating to interact with different dermal MNP subtypes will also be true for Epi-cDC2s, although the target cells may differ, leading to subsequent differences in T cell stimulation, a point which is currently being investigated with human foreskin explant models. Apoptosis mediated by early HSV proteins while they migrate may protect the rest of the epidermal/dermal MNP network from viral spread, as we previously detected no viral spread into the recipient dermal MNPs taking up infected LCs [27] and is consistent with the low extracellular virus concentrations produced by the epidermal MNPs, as shown here. However viral replication, now demonstrated within these cells, provides a full repertoire of structural and nonstructural viral antigens for initiating the immune response through the relay.

**Fig 10:**
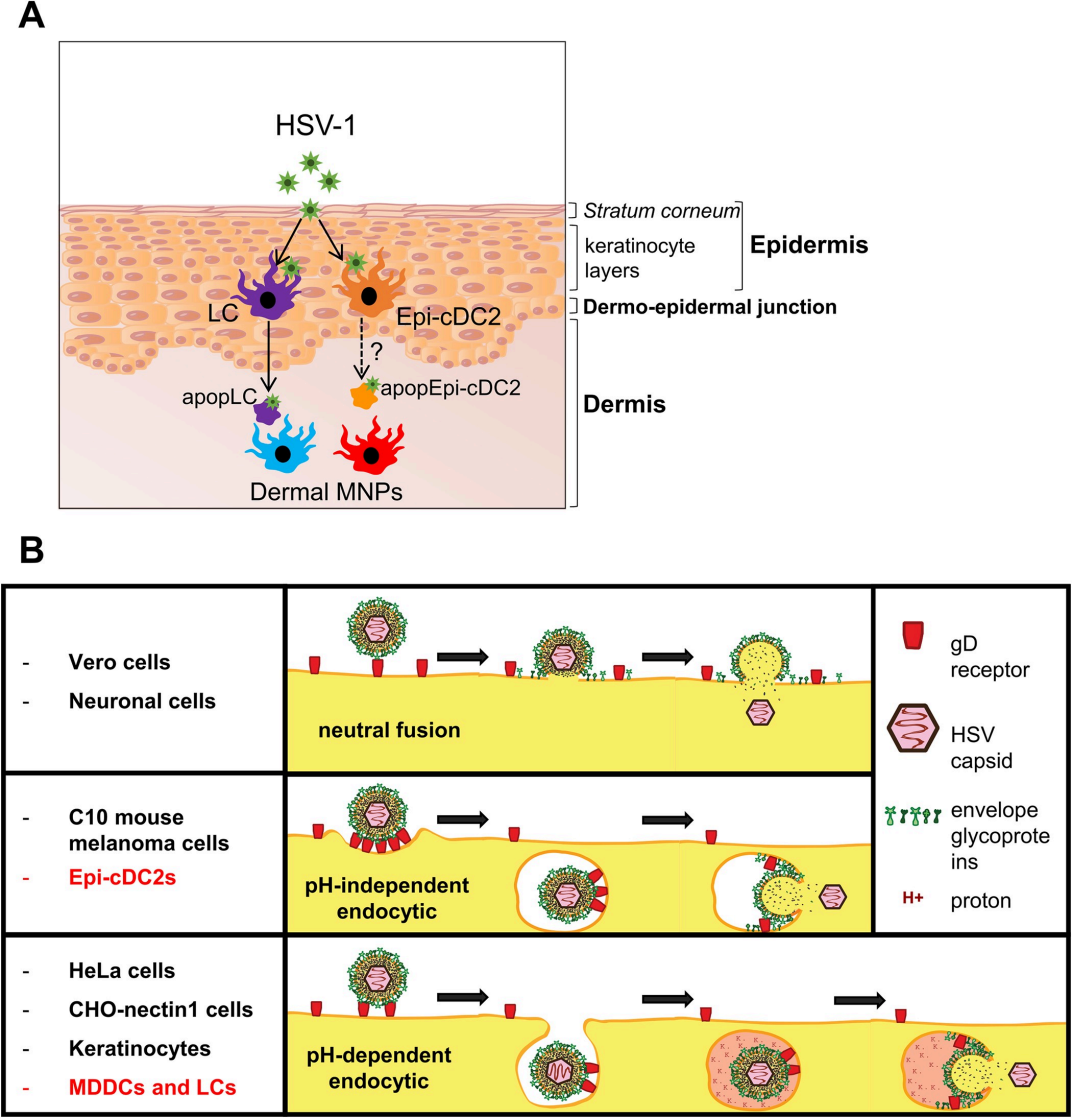
The HSV viral relay between epidermal and dermal MNPs and HSV entry pathways into them compared to other target cells. **(A)** HSV enters skin where the *stratum corneum* is damaged or absent and infects LCs and Epi-cDC2s, located in the epidermis. Upon HSV infection both cell types undergo apoptosis. HSV infected LCs migrate to the dermis, where they cluster with and are taken up by dermal MNPs, which may go on to present the HSV antigens to T cells. Whether HSV infected apoptotic Epi-cDC2s also migrate to the dermis and are taken up by dermal MNPs, thereby contributing to the HSV viral relay, is being investigated. **(B)** HSV enters cells via different pathways depending on the host cell type. HSV enters Vero and neuronal cells by neutral fusion at the plasma membrane. A pH-independent endocytic pathway is utilised for C10 mouse melanoma cells and Epi-cDC2s, whereas a pH-dependent endocytic pathway is utilised in epithelial cells such as keratinocytes, HeLa cells and CHO-nectin 1 cells, as well as predominantly in human MDDCs and LCs.

There are potential implications of the uptake of apoptosing LCs and presumably Epi-cDC2s by different dermal MNP subsets. In human skin, cDC1s are more efficient than other MNP subsets at ‘cross-presentation’ of exogenous antigens and readily take up apoptosing cells. So uptake of LCs and Epi-cDC2s may lead to HSV specific CD8^+^ T cell stimulation, most likely initially in lymph node but perhaps also locally in skin. Presumably, the other MNPs, including cDC2s and monocyte-derived macrophages present ‘exogenous HSV antigens’ or those processed through the MHC class II pathway directly to stimulate CD4^+^ T cells, as in mice [8,9,64]. Our results also suggest that, as for HIV, the HSV interaction with and uptake by Epi-cDC2s is a second pathway for initiation of the immune response to HSV in the epidermis and dermis, as shown in summary Fig 10.

There are two main practical implications for these results. Firstly, prior genital HSV-2 infection enhances sexual HIV acquisition 3–5 fold (especially important in Africa/India) [65]. The major target cells for HIV in foreskin are epidermal LCs or cDC2s, and in the dermis, resting memory CD4^+^ T cells and macrophages. Anogenital LCs are major target cells for infection by both viruses [27,50,66,67]. cDC2 are also a major early target for SIV in macaque models [68,69]. We have now shown that both HIV and HSV can infect epidermal LCs and Epi-cDC2s, so they may be sites for synergistic infection in the same or, more likely, adjacent cells and/or act as a facilitated conduit for HIV to transfer to dermal CD4^+^ T cell targets.

Secondly these pathways have implications for designing vaccines to target the appropriate MNPs in anogenital mucosa. A successful prophylactic vaccine against initial genital herpes must prevent or markedly reduce local spread in the skin/mucosa and also HSV seeding of the neuronal ganglia via the cutaneous sensory nerves to impact on the frequency of subsequent recurrences. This might be achieved by inducing resident CD8^+^ (or CD4^+^) T cells, which can quickly migrate into the epidermis and kill infected cells or produce rapidly diffusing antiviral cytokines upon infection. Modelling of the immune control and resolution of genital herpes lesions strongly suggests that CD8^+^ T cell stimulation is important for HSV control, probably with CD4^+^ T cell help [70,71], and these are likely to be primed by different DC subsets.

Understanding the roles of specific human MNP subsets in response to HSV infection should guide the targeting of vaccines (and adjuvants) to specific subsets, perhaps simulating the same immune responses as natural infection, to induce the recruitment and stimulation of local resident memory CD8^+^ T cells. Recruitment of B and T cells from the blood may be too slow to prevent significant local spread and viral seeding of the nerves.

## Materials and methods

### Ethics statement

This study was approved by the Ethics Committee for Western Sydney Local Health District, approval no. 4192–2019/ETH01894. Written informed consent was received from participants or legal guardians of infants or young children aged under 14 prior to inclusion in this study.

### Virus preparation

HSV-1 strain F expressing GFP-US9 (HSV-1 GFP) was kindly provided by Prof Renato Brandimarti [72]. In some experiments HSV-1 strain F was used. The virus stocks were prepared by infecting 80–90% confluent monolayers of Vero cells incubated in DMEM (Lonza, USA) supplemented with 2% FBS (Sigma-Aldrich, USA). After two days, cells were harvested, freeze-thawed three times, and briefly pulse-sonicated using a cup horn sonicator (Branson, USA) to release cell-associated virus. Debris was removed by centrifugation at 450 x g for 10 minutes (min) at 4°C and supernatant was stored at -80°C until use. Virus titres were determined by plaque assay as previously described [23]. Briefly, 10-fold dilutions of the virus were inoculated on a confluent monolayer of Vero cells for 1 h at 37°C. The inoculum was removed and fresh medium containing 1.5% w/v Carboxymethylcellulose sodium salt (CMC, medium viscosity, Sigma-Aldrich) was added. After 2 days, cells were fixed and stained with 0.1% crystal violet solution to count the number of plaques.

### Development of HaCaT-conditioned media

HaCaT cells were cultured in DMEM supplemented with 10% FBS at 37°C, 5% CO_2_ and passaged every 3 days once they reached 80–90% confluency. To obtain HaCaT-conditioned media, for the final passage, once cell cultures reached approximately 80% confluency the culture media was removed and cells were washed twice with PBS. The media was replaced with “supplemented AIM V medium”: AIM V serum-free medium (Gibco, USA) supplemented with 10 μM HEPES (Gibco), 1 μM sodium pyruvate (Gibco), 1 x non-essential amino acids (Gibco), 50 μM 2-Mercaptoethanol (Gibco) and 10 μg/mL gentamicin (Gibco). The HaCaT cells were cultured for an additional 2 days in this medium, after which the medium was harvested and filter-sterilised through a 0.22 μm syringe-driven filter unit. The HaCaT-conditioned medium was then stored in aliquots at -20°C until required.

### Tissue processing and isolation of epidermal cells

Abdominal skin was collected immediately after surgery and processed as previously described [35]. Briefly, skin pieces were removed from underlying tissue using a skin graft knife (Swann-Morton, UK), and the resulting skin grafts were passed through a skin graft mesher (Zimmer Biomet, USA). To facilitate the mechanical separation of the epidermis from underlying dermis, the meshed skin pieces were placed in RPMI 1640 (Lonza) containing 0.14 U/mL dispase (Worthington Industries, USA) and 50 μg/mL gentamicin, and rotated at 4°C overnight. The skin was then washed in PBS and epidermis was separated from dermis using fine forceps. To liberate epidermal cells, epidermal tissue was incubated with RPMI 1640 containing 200 U/mL collagenase Type IV (Worthington) at 37°C for 120 min. A tea strainer was used to separate the cells from undigested tissue, and then the supernatants were passed through a 100 μm cell strainer (Greiner Bio-One, Germany). The epidermal cells were pelleted, washed twice with cold PBS and resuspended in RPMI 1640 for centrifugation on a Ficoll-Paque PLUS (GE Healthcare, USA) density gradient at 450 x g for 20 min to harvest the epidermal MNPs. For Caspase 3 experiments, a Dead Cell Removal kit (StemCell Technologies, Canada), was used as per manufacturer’s directions to remove dead cells prior to culture.

### Generation of monocyte-derived MNPs

Fresh PBMC were isolated from human whole blood obtained from the Sydney Red Cross Blood Bank, and CD14+ monocytes were obtained by positive selection using CD14 microbeads (Miltenyi Biotec, Germany) as previously described [23]. To generate immature MDDCs, CD14^+^ monocytes were cultured in RPMI1640 supplemented with 10% FBS, as well as IL-4 (500 IU/mL) and GM-CSF (300 IU/mL) (Life technologies, USA) for 5–6 days at 37°C, 5% CO_2_ as previously described [34].

### *In vitro* HSV infection and culture

Isolated epidermal MNPs were inoculated with HSV-1 GFP, HSV-1, or with RPMI 1640 only for mock treatment for 1 h at 37°C at a MOI of 10. MDDCs, Vero and HeLa cells were inoculated at a MOI of 3. Cells were washed with RPMI 1640 and then cultured for the time points specified in individual experiments. Vero and HeLa cells were cultured in DMEM supplemented with 5% FBS, MDDCs were cultured in RPMI 1640 supplemented with 10% FBS, while epidermal cells were cultured in HaCaT conditioned medium, described previously. After culture, Vero and HeLa cells were detached from the culture flask using cell-dissociation buffer (Life Technologies) for 30 min. All cell types were washed twice with PBS and prepared for analysis by flow cytometry. To detect extracellular virus in the cell culture media of HSV-infected LCs and Epi-cDC2s, epidermal MNPs were FACS sorted (see Flow Cytometry) into LCs and Epi-cDC2s and inoculated with HSV-1 GFP at an MOI of 10 for 1 h at 37°C. The inoculum was washed off three times and cells resuspended at a concentration of 7x10^4^/mL in HaCaT conditioned medium. At the end of the culture time, cells were pelleted at 350 xg for 5 min, the supernatant was harvested and an additional centrifugation step at 2500 xg for 10 min was performed. Supernatant was aliquoted and stored at -80°C. Extracellular virus was detected in the supernatants by fluorescent plaque assay performed on Vero monolayers in Nunc Lab-Tek II 8 well Chamber Slides (ThermoFisher, USA). After 40 h, culture media was removed and cells were fixed with absolute methanol for 10 mins, then left to air dry. Chambers were removed with the provided device and coverslips were mounted to the slides using Fluoromount-G Mounting Medium (Invitrogen, USA). Fluorescent plaques were counted from 10x images obtained using an Olympus VS120 Virtual Slide Microscope.

### HSV entry and inhibition studies

Inhibitors were purchased from Sigma-Aldrich unless otherwise specified. For experiments using acyclovir (acycloguanosine), cells were inoculated with HSV-1 or mock treated for 1 h at 37°C as described in the previous section. Cells were washed with PBS and a Dead Cell Removal kit (StemCell Technologies) was used. Cell were then cultured in HaCaT conditioned media in the presence of the acyclovir (500 μg/mL) at 37°C for 18 h. Monensin (BD GolgiStop) was from BD Biosciences (USA), and mouse anti-human langerin/CD207 blocking antibody (817G7) was from Novus Biologicals (USA). For experiments using bafilomycin A (baf A), monensin or mouse anti-human langerin antibody: isolated epidermal MNPs, MDDCs, Vero or HeLa cells were pre-treated in serum-free media (AIM-V media for epidermal cells, RPMI 1640 for MDDCs, DMEM for Vero and HeLa cells) in the absence or presence of inhibitor for 30 min at 37°C at the concentrations specified. The cells were then inoculated with HSV-1 or mock treated for 1 h at 37°C as described in the previous section, with the continued presence of the inhibitor in the media. Cells were washed with serum-free media and cultured in the presence of the inhibitor at 37°C for the times specified. Cells were then washed twice with PBS and prepared for analysis by flow cytometry. For experiments using methyl-β-cyclodextrin (mβCD), cytochalasin D (cyto D) and latrunculin A (lat A): after pre-treatment with the inhibitor as described, the cells were inoculated with HSV-1 or mock treated for 1 h at 4°C, followed by washing, resuspending in media and incubating for an additional 1 h at 37°C with the continued presence of the inhibitor as has been done previously [40]. The cells were washed with PBS and incubated with 0.5% trypsin for 15 min at 37°C, then washed twice with PBS and prepared for analysis by flow cytometry.

### Flow cytometry

Cells were labelled in aliquots of 2.5 x 10^6^ cells per test in 50 μL of buffer as previously described (Bertram et al., 2019). Antibody incubations were conducted for 30 min at 4°C. To exclude nonviable cells, cells were stained with LIVE/DEAD Fixable Aqua or Near-IR Dead Cell Stain Kit (Life Technologies). For the epidermal cells, antibodies used to distinguish LCs and Epi-cDC2s according to cell surface phenotype were: HLA-DR BUV395 (G46-6, BD Horizon), CD45 PE-Cy7 (HI30, BD Pharmingen), CD1a BV510 (HI149, BD Horizon), CD11c PE-CF594 (B-LY6, BD Horizon), and Langerin Vioblue (MB22-9F5, Miltenyi Biotec). The gating strategy used to define LCs and Epi-cDC2s is shown in Fig 2A. Antibodies used to label surface markers of interest were CD270 PE (HVEM, 122, BioLegend, USA), CD111 PE (nectin-1, R1.302, BioLegend), the isotype control IgG1 PE (MOPC-21, BioLegend), and HSV-1 glycoprotein C(gC)-FITC (Virostat, USA). To detect intracellular ICP27, a mouse anti-HSV-1 & 2 nuclear antibody (0119, Virostat) was Biotin conjugated using the Mix-n-Stain Antibody Labeling Kit (Biotium, USA). The antibody was concentrated using the Biotium columns and the conjugation was performed according to the manufacturer’s protocol and transferred to storage buffer. To detect intracellular active Caspase-3, ICP27 or gM, intracellular staining was performed following LIVE/DEAD Fixable Near-IR and surface antibody labelling. Cells were incubated with BD Cytofix/Cytoperm solution for 15 min at room temperature, washed with a permeabilization buffer (1% BSA (w/v), 0.1% saponin (w/v), 0.1% sodium azide (w/v) in PBS) and blocked with 10% human AB serum (Sigma-Aldrich) for 10 min at room temperature. Rabbit anti-Active Caspase-3 (C92-605, BD Pharmingen), or rabbit-anti-gM from Thomas Mettenleiter [73], was then incubated at room temperature for 30 min. Cells were washed three times with permeabilization buffer then blocked with 10% human AB serum for 10 min at room temperature. Alexa Fluor-647 conjugated goat anti-rabbit IgG (Life Technologies) was added and cells were then incubated at room temperature for 30 min. Cells were washed three times with permeabilization buffer. Intracellular staining was performed as above for Biotin conjugated anti-ICP27 followed by Alexa Fluor-647 conjugated streptavidin (Life Technologies). Flow cytometry was performed on the BD LSRFortessa flow cytometer and data was analysed using FlowJo (version 10, BD).

For sorting, cells were as above, stained with LIVE/DEAD Fixable Near-IR, HLA-DR PerCP (G46-6, BD Horizon), CD45 PE (HI30, BD Pharmingen), CD1a BV510, and CD11c PE-CF594. FACS was performed on a BD FACSAria III (100 μm nozzle and 20 pounds/square inch). Sorted cells were collected into FACS tubes containing RPMI with 10% human AB serum.

### qPCR

To measure HSV-1 ICP4 gene expression in MDDCs, RNA was extracted using the RNAqueous kit (Ambion, USA). RNA was DNase I treated (Life Technologies) and reverse transcribed using oligo(dT) and SuperScript III RNAse H reverse transcriptase (Life Technologies). HSV-1 ICP4 was detected by the following primer pair: Fwd: 5′-CGACACGGATCCACGACCC-3′; Rev: 5′-GATCCCCCTCCCGCGCTTCGTCCG-3′ (Sigma-Aldrich) using Platinum SYBR Green qPCR Supermix-UDG (Life Technologies) on a Mx3005P machine (Stratagene, Agilent Technologies, USA). The following cycling conditions were used: 50°C for 2 min, 95°C for 2 min, 45 cycles of 95°C for 15 sec, 55°C for 30 sec and 72°C for 30 sec. The relative quantitation method (ΔΔC_T_) [74] was used to evaluate the expression of selected genes, normalised to glyceraldehyde-3-phosphate dehydrogenase (GAPDH). Data was analysed using MxPro QPCR software (version 4).

### *Ex vivo* inner foreskin explants

Healthy foreskin tissues were obtained from three donors aged 5, 9 and 12 years old undergoing circumcision and processed similarly to as previously described [35]. Briefly, inner foreskin was separated from outer foreskin and 1x10^7^ PFU of HSV-1 GFP (diluted in RPMI 1640 supplemented with 50g/mol sucrose and 0.05% Poloxamer 188, Sigma-Aldrich) or RPMI 1640 only as a mock-infected control were topically applied to the tissue surface. The explants were mounted on Gelfoam absorbable gelatin sponges (Pfizer, USA) in a 24-well plate, soaked in RPMI 1640 supplemented with 10% FBS, 10 μm HEPES, 1 mM sodium pyruvate, 1x non-essential amino acids, 50 μM 2-Mercaptoethanol and 10 μg/mL gentamicin (Gibco), and cultured for 24 h at 37°C. After culture, tissue explants were rinsed with PBS, and tissues were snap-frozen in Tissue-Tek O.C.T compound (ProSciTech, Kirwan, Australia) and stored at -80°C. Samples were then cryosectioned into 7 μm sections and stored at -80°C until immunofluorescence labelling was performed.

### Immunofluorescence labelling and microscopy

Sectioned slides were briefly thawed at room temperature, then fixed with 2% PFA diluted in PBS for 20 min, followed by blocking for 30 min at room temperature (10% normal donkey serum, 1% BSA and 0.1% saponin diluted in PBS). Primary and secondary antibodies were applied and incubated at 37°C for 45 min and 30 min respectively. Antibodies used include rabbit anti-GFP (polyclonal, Abcam, UK), goat anti-langerin (polyclonal, R&D Systems, USA), mouse anti-CD11c (3.9, eBioscience, USA), Alexa Fluor-488 conjugated donkey anti-rabbit IgG, Alexa Fluor-546 conjugated donkey anti-mouse IgG and Alexa Fluor-647 conjugated donkey anti-goat IgG (Life Technologies). Following this, the Vector TrueVIEW Autofluorescence Quenching Kit (Vector Laboratories, USA) was applied to each slide according to the manufacturer’s instructions for 1.5 min then rinsed with PBS. Coverslips were mounted to the slides using ProLong Diamond Antifade Mountant with DAPI (Life Technologies). Images were acquired using a 20x objective on an Olympus VS120 Virtual Slide Microscope and the Olympus VS-ASW 2.9 software, followed by subsequent analysis in Fiji (Image J version 2.0.0-rc-65/1.51w). High power z-stack images were acquired using a 60x objective on a DeltaVision restoration microscope with a Photometrics CoolSnap QE camera (GE Healthcare) and deconvolved using DeltaVision softWoRx software suite (version 6.5.2). Subsequent analysis of orthogonal views was performed in Fiji.

### Statistical analysis

Graphpad Prism (version 8) was used to perform statistical analyses and generate graphs. Data are described as mean ± standard deviation (S.D.) unless stated otherwise. Two-tailed paired t-tests were used to analyse two-sample paired data (Figs 2C, 3A and 5). For unpaired data with three or more groups, one-way (Fig 8E) or two-way ANOVAs (Fig 6B) with Tukey’s test for multiple pairwise comparisons were used to analyse differences in one or two variables respectively. Repeated measures one-way ANOVA with Tukey’s test for multiple pairwise comparisons was used to analyse differences in one variable for paired data with three or more groups (Figs 4B, 7, 8 and 9). Repeated measures one-way ANOVA with Bonferroni’s test for multiple comparisons was used to analyse pairwise differences in one variable compared to a control group (Fig 6C). Repeated measures two-way ANOVA was used to analyse differences in two variables with Bonferroni’s test for multiple comparisons used to analyse pairwise differences in either variable (Fig 2E). For statistical significance, * = p<0.05, ** = p<0.01, *** = p<0.001, **** = p<0.0001. Where practical, exact p values are shown in the figures, or otherwise they are included in the figure legends.

## Supporting information

S1 Fig Additional interactions of LCs and Epi-cDC2s with HSV-1 infected keratinocytes in inner foreskin explants. Inner foreskin explants were topically infected with HSV-1 GFP or mock infected for 24 h. Images show LCs (langerin^+^ CD11c^-^, blue cells) and Epi-cDC2s (CD11c^+^Langerin^+/-^, red and red/blue dual labelled cells) in the epidermis of HSV-1 GFP infected (green) foreskin explants at 20x magnification. Epi-cDC2s and LCs interacting with HSV-1 GFP infected keratinocytes are marked by yellow and white arrows respectively in **(A)** images taken from different tissue sections of the 9 y.o. (left panel) and 5 y.o. (right panel) donors shown in Fig 1 and **(B)** a representative image from an additional 12 y.o. donor. Scale bars indicate 50 μm.
(TIF)

S2 Fig Cell viability in the presence of bafilomycin A is comparable to HSV infection alone. **(A)** Day 6 MDDCs were pre-treated for 30 min at 37°C with bafilomycin A (baf A), at the concentrations shown. HSV-1 (strain F) was added (MOI of 3) in the continued presence of baf A and allowed to bind for 1 h at 37°C. Cells were then washed and incubated in the presence of inhibitors at 37°C for a total infection time of 18 h. Cells were washed with PBS and stained with fixable LIVE/DEAD Aqua and an HSV-1 gC-FITC antibody then analysed by flow cytometry. The percentage of live cells was calculated by the percentage of intact cells in the FSC-SSC gate multiplied by the percentage of cells negative for the LIVE/DEAD Aqua stain. **(B)** A mixed population of epidermal MNPs was isolated from human abdominal epidermis, pretreated with baf A (100 nM) and inoculated with HSV-1 GFP (MOI of 10) or mock treated for 1 h, then washed and incubated in the presence of baf A (100 nM) for a total infection time of 18 h in HaCaT-conditioned medium at 37°C. The cells were then washed with PBS and labelled with fixable LIVE/DEAD Near-IR followed by antibodies to HLA-DR, CD45, CD1a, CD11c, and Langerin. The percentage of live cells of each subset is shown as representative plots (left). The percentage of HSV-1 GFP^+^ cells at 18 h in LCs (purple) and Epi-cDC2s (orange) is shown in box and whisker plots (right), n = 6.
(TIF)

S3 Fig Comparative levels of apoptosis in HSV-1 GFP+ and bystander LCs and Epi-cDC2s. Additional analysis of the experiments in Fig 8. Within the HSV-1 GFP infected condition, LCs and Epi-cDC2s were each gated into HSV-1 GFP^+^ and bystander populations as shown in Fig 8A. Data show the percentage of cells, HSV-1 GFP^+^ or bystander, in each apoptosis quadrant for LCs and Epi-cDC2s in box and whisker plots. Repeated measures one-way ANOVAs with Tukey’s multiple comparisons were used to compare the percentage of HSV-1 GFP^+^ or bystander LCs and Epi-cDC2s in the apoptosis quadrants, n = 4.
(TIF)

S1 Data This file contains the source data used to generate all graphs in this manuscript (including supplemental figures). Each tab of the Excel file contains the data for a separate figure and data are grouped into panels as presented in the figures.
(XLSX)
