# Supplementary figures and images for: Herpes Simplex Virus type 1 infects Langerhans cells and the novel epidermal dendritic cell, Epi-cDC2s, via different entry pathways
Source: PLoS Pathog. 2021 Apr 27;17(4):e1009536. doi: 10.1371/journal.ppat.1009536 (PMC8104422; doi:10.1371/journal.ppat.1009536)

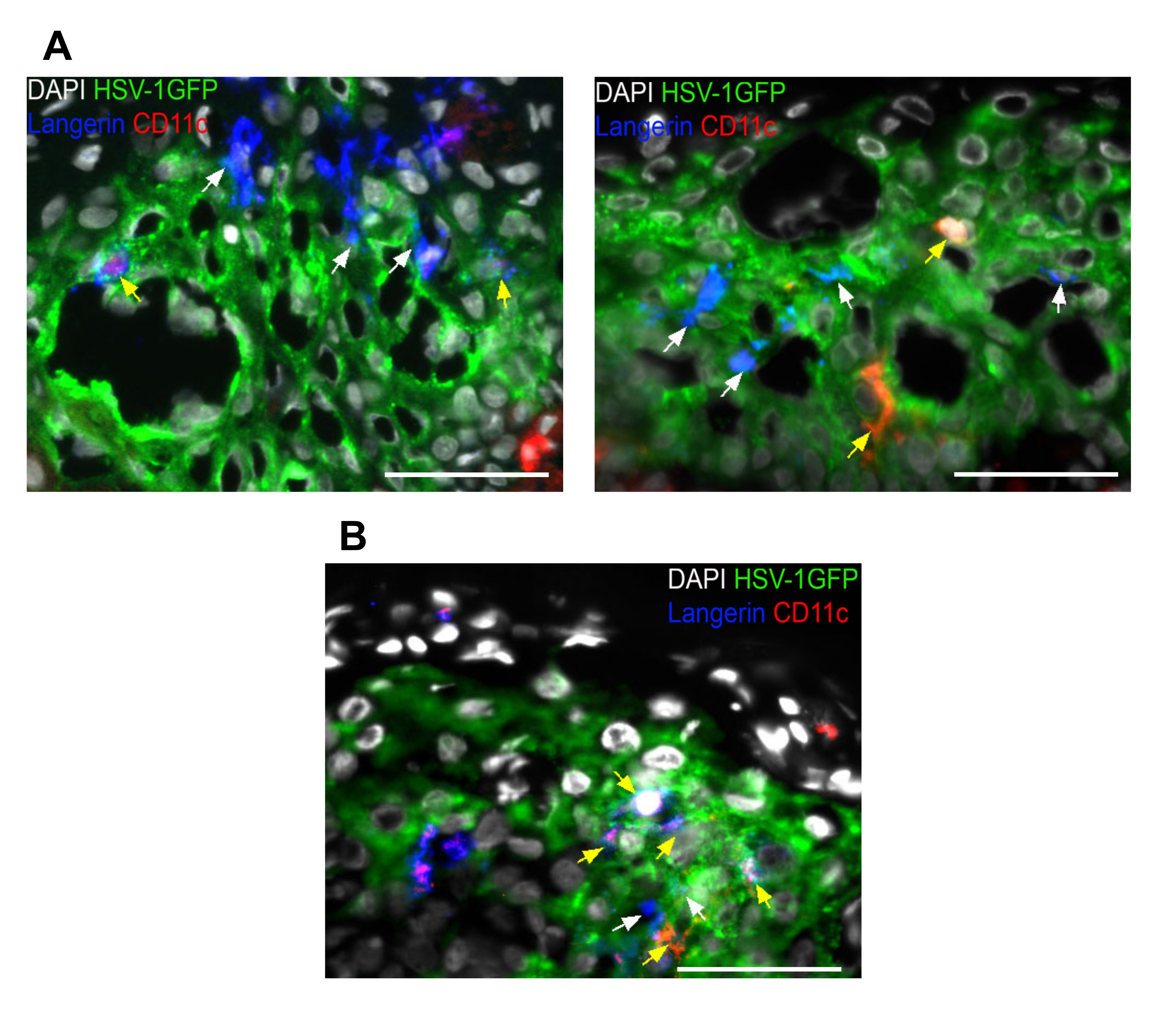

Supplement: S1 Fig — Inner foreskin explants were topically infected with HSV-1 GFP or mock infected for 24 h. Images show LCs (langerin+ CD11c-, blue cells) and Epi-cDC2s (CD11c+Langerin+/-, red and red/blue dual labelled cells) in the epidermis of HSV-1 GFP infected (green) foreskin explants at 20x magnification. Epi-cDC2s and LCs interacting with HSV-1 GFP infected keratinocytes are marked by yellow and white arrows respectively in (A) images taken from different tissue sections of the 9 y.o. (left panel) and 5 y.o. (right panel) donors shown in Fig 1 and (B) a representative image from an additional 12 y.o. donor. Scale bars indicate 50 μm. (TIF) [file ppat.1009536.s001.tif]

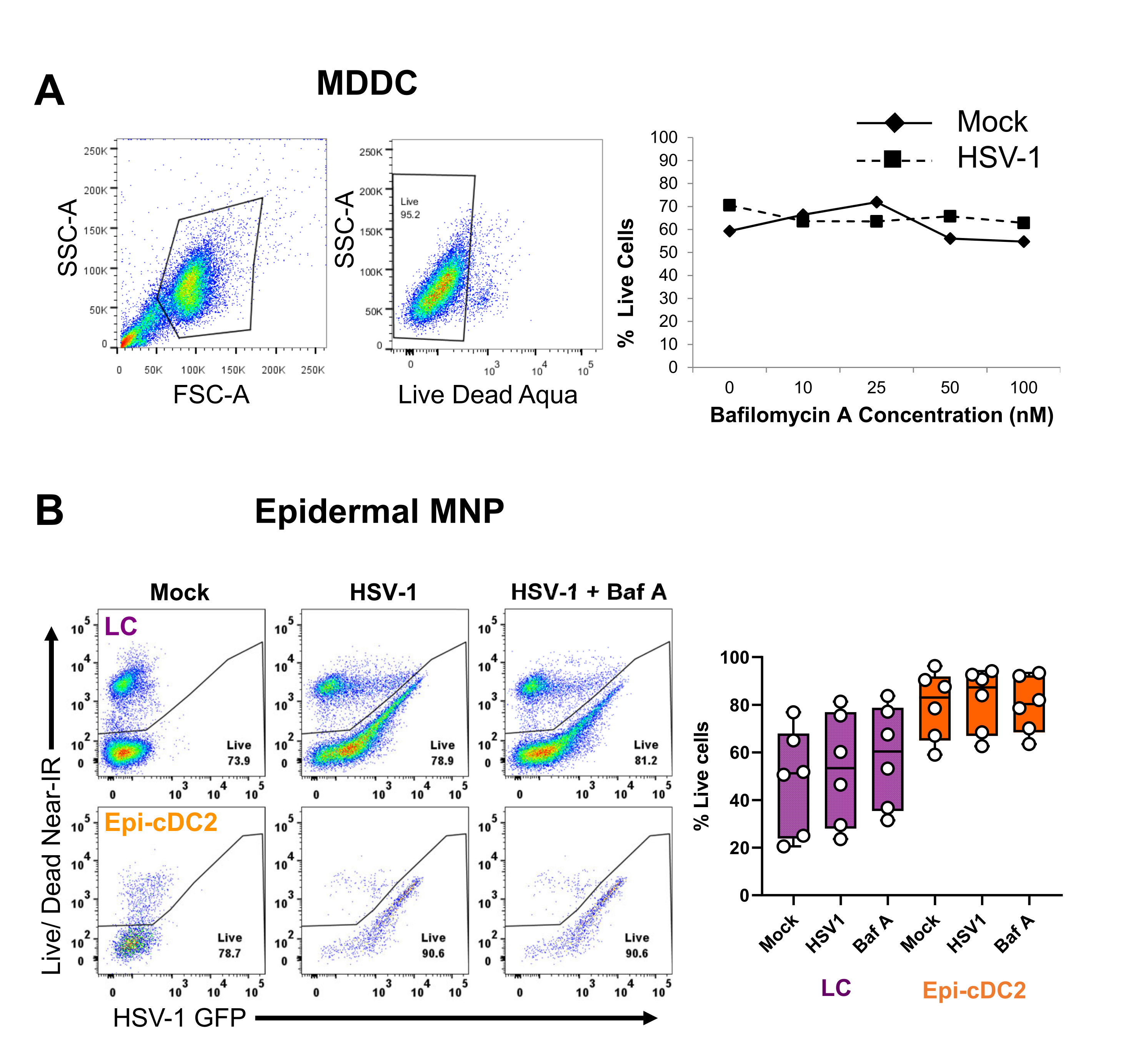

Supplement: S2 Fig — (A) Day 6 MDDCs were pre-treated for 30 min at 37°C with bafilomycin A (baf A), at the concentrations shown. HSV-1 (strain F) was added (MOI of 3) in the continued presence of baf A and allowed to bind for 1 h at 37°C. Cells were then washed and incubated in the presence of inhibitors at 37°C for a total infection time of 18 h. Cells were washed with PBS and stained with fixable LIVE/DEAD Aqua and an HSV-1 gC-FITC antibody then analysed by flow cytometry. The percentage of live cells was calculated by the percentage of intact cells in the FSC-SSC gate multiplied by the percentage of cells negative for the LIVE/DEAD Aqua stain. (B) A mixed population of epidermal MNPs was isolated from human abdominal epidermis, pretreated with baf A (100 nM) and inoculated with HSV-1 GFP (MOI of 10) or mock treated for 1 h, then washed and incubated in the presence of baf A (100 nM) for a total infection time of 18 h in HaCaT-conditioned medium at 37°C. The cells were then washed with PBS and labelled with fixable LIVE/DEAD Near-IR followed by antibodies to HLA-DR, CD45, CD1a, CD11c, and Langerin. The percentage of live cells of each subset is shown as representative plots (left). The percentage of HSV-1 GFP+ cells at 18 h in LCs (purple) and Epi-cDC2s (orange) is shown in box and whisker plots (right), n = 6. (TIF) [file ppat.1009536.s002.tif]

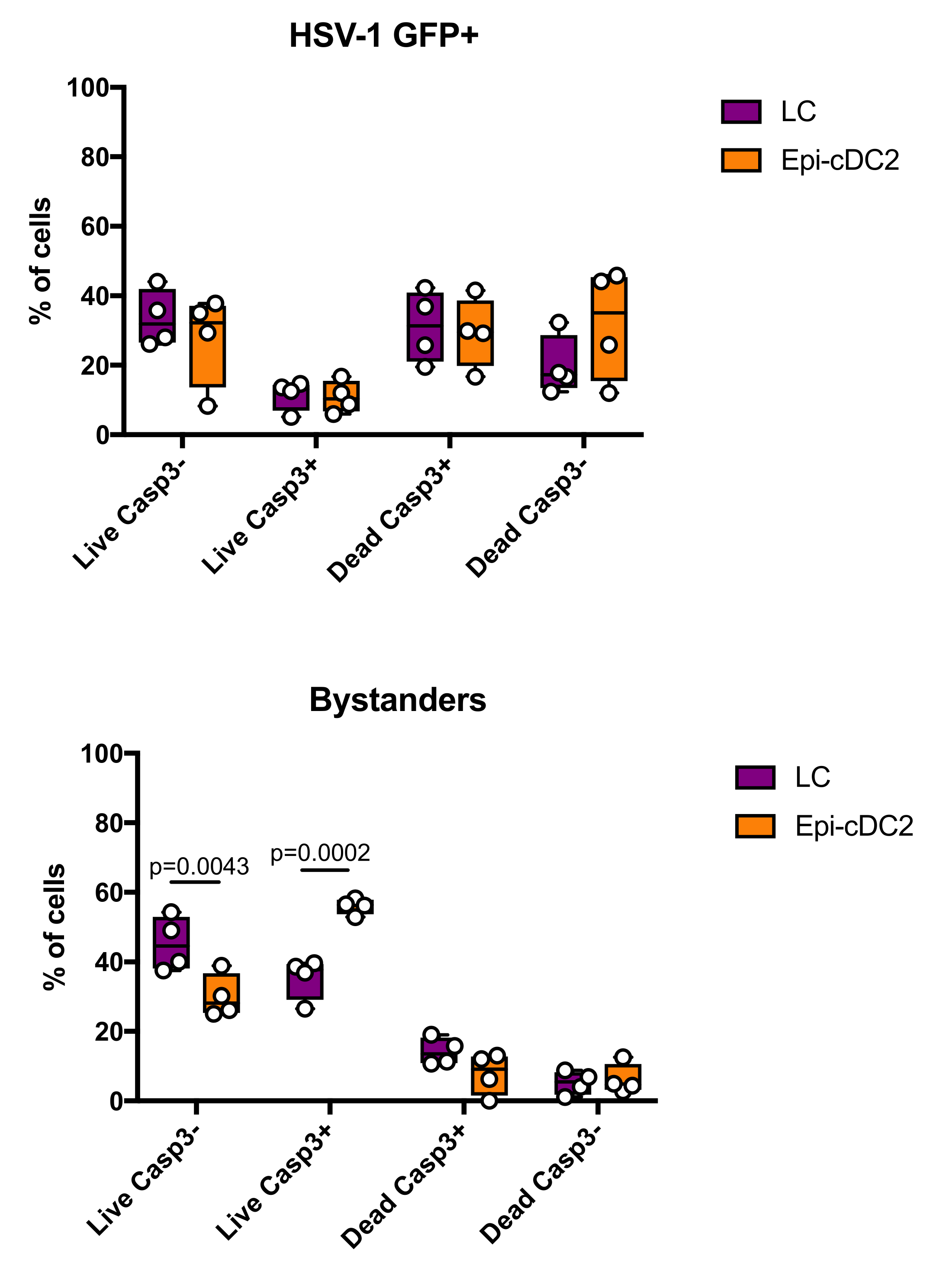

Supplement: S3 Fig — Additional analysis of the experiments in Fig 8. Within the HSV-1 GFP infected condition, LCs and Epi-cDC2s were each gated into HSV-1 GFP+ and bystander populations as shown in Fig 8A. Data show the percentage of cells, HSV-1 GFP+ or bystander, in each apoptosis quadrant for LCs and Epi-cDC2s in box and whisker plots. Repeated measures one-way ANOVAs with Tukey’s multiple comparisons were used to compare the percentage of HSV-1 GFP+ or bystander LCs and Epi-cDC2s in the apoptosis quadrants, n = 4. (TIF) [file ppat.1009536.s003.tif]
